# Potential Role of Chronic Physical Exercise as a Treatment in the Development of Vitiligo

**DOI:** 10.3389/fphys.2022.843784

**Published:** 2022-03-10

**Authors:** Elias de França, Ronaldo V. T. dos Santos, Liliana C. Baptista, Marco A. R. Da Silva, André R. Fukushima, Vinícius B. Hirota, Raul A. Martins, Erico C. Caperuto

**Affiliations:** ^1^Human Movement Laboratory, São Judas University, São Paulo, Brazil; ^2^Departamento de Biociências, Universidade Federal de São Paulo, São Paulo, Brazil; ^3^Faculty of Sport, Research Centre in Physical Activity, Health and Leisure, University of Porto, Porto, Portugal; ^4^Center for Exercise Medicine, University of Alabama at Birmingham, Birmingham, AL United States; ^5^Targeted Exercise, Microbiome and Aging Laboratory, University of Alabama, Birmingham, AL United States; ^6^Faculty of Sport Sciences and Physical Education, University of Coimbra, Coimbra, Portugal; ^7^Department of Physical Education, Universidade da Amazônia, Belém, Brazil; ^8^Centro Universitário das Américas – FAM, São Paulo, Brazil; ^9^Faculdade de Ciências da Saúde – IGESP – FASIG, São Paulo, Brazil

**Keywords:** vitiligo, autoimmune disease, physical training, immune system, oxidative stress, metabolic syndrome

## Abstract

*Vitiligo* is an autoimmune disease characterized by progressive skin depigmentation and the appearance of white patches throughout the body caused by significant apoptosis of epidermal melanocytes. Despite not causing any physical pain, vitiligo can originate several psychosocial disorders, drastically reducing patients’ quality of life. Emerging evidence has shown that vitiligo is associated with several genetic polymorphisms related to auto-reactivity from the immune system to melanocytes. Melanocytes from vitiligo patients suffer from excess reactive oxygen species (ROS) produced by defective mitochondria besides a poor endogenous antioxidant system (EAS). This redox imbalance results in dramatic melanocyte oxidative stress (OS), causing significant damage in proteins, lipid membranes, and DNA. The damaged melanocytes secret damage-associated molecular pattern (DAMPs), inducing and increasing inflammatory gene expression response that ultimately leads to melanocytes apoptosis. Vitiligo severity has been also associated with increasing the prevalence and incidence of metabolic syndrome (MetS) or associated disorders such as insulin resistance and hypercholesterolemia. Thus, suggesting that in genetically predisposed individuals, the environmental context that triggers MetS (i.e., sedentary lifestyle) may also be an important trigger for the development and severity of vitiligo disease. This paper will discuss the relationship between the immune system and epidermal melanocytes and their interplay with the redox system. Based on state-of-the-art evidence from the vitiligo research, physical exercise (PE) immunology, and redox system literature, we will also propose chronic PE as a potential therapeutic strategy to treat and prevent vitiligo disease progression. We will present evidence that chronic PE can change the balance of inflammatory to an anti-inflammatory state, improve both EAS and the mitochondrial structure and function (resulting in the decrease of OS). Finally, we will highlight clinically relevant markers that can be analyzed in a new research avenue to test the potential applicability of chronic PE in vitiligo disease.

## Introduction

Vitiligo is an autoimmune disease characterized by progressive skin depigmentation and the appearance of white patches throughout the body, caused by significant apoptosis of epidermal melanocytes (Bergqvist and Ezzedine, 2020). Currently, it is estimated that vitiligo affects 0.5–2% of the global population, being the most prevalent skin disease (Krüger and Schallreuter, 2012), and despite not causing any physical pain, vitiligo can generate several psychosocial disorders reducing drastically patients’ quality of life (Krüger and Schallreuter, 2013).

There is no known cure for vitiligo, but there are several management strategies to reduce the spread of white skin patches and to attempt to re-pigmentate the affected areas. The most used treatments are immunosuppressive drugs such as corticosteroids, calcineurin inhibitors, antioxidant supplements, and phototherapies. However, these therapeutics are not 100% effective and do not prevent the disease reappearance (Bergqvist and Ezzedine, 2020). Therefore, it is still needed cost-effective strategies to prevent the resurgence of vitiligo wounds and effectively stop the spread of white patches. Knowing the underlying molecular mechanisms and patients ‘environmental context is essential to develop effective treatments.

Emerging evidence has shown that vitiligo is associated with several genetic polymorphisms related to auto-reactivity from the immune system (IS) to melanocytes. Melanocytes from vitiligo patients suffer from excess reactive oxygen species (ROS) produced by defective mitochondria besides a poor endogenous antioxidant system (EAS) (Bergqvist and Ezzedine, 2020). This redox imbalance results in dramatic melanocyte oxidative stress (OS), causing significant damage in proteins, lipid membranes, and DNA. The damaged melanocytes secret damage-associated molecular pattern (DAMPs), inducing and increasing inflammatory gene expression response that ultimately leads to cell apoptosis (Bergqvist and Ezzedine, 2020).

Vitiligo severity has also been associated with metabolic syndrome (MetS), increasing the prevalence and incidence of MetS or associated disorders such as insulin resistance and hypercholesterolemia (Ataş and Gönül, 2017; Sharma et al., 2017; Tanacan and Atakan, 2020; Verma et al., 2021). Etiologically, MetS are associated with a sedentary lifestyle (Edwardson et al., 2012), cellular inflammation, and OS mechanisms (Bonomini et al., 2015) that may be involved in the onset of vitiligo. Therefore, we speculate that in genetically predisposed individuals, the environmental context that triggers MetS (i.e., sedentary lifestyle) may also be an important trigger for the development and severity of vitiligo disease.

In this paper, we will discuss the molecular mechanisms and the role of patients’ environmental context for the onset of vitiligo. Specifically, we will discuss the relationship between the immune system and epidermal melanocytes and their interplay with the redox system. Based on state-of-the-art evidence from the vitiligo research, physical exercise (PE) immunology, and redox system literature, we also propose *PE* as a potential therapeutic strategy to fight vitiligo adverse events (e.g., spreading new white skin wounds and associated comorbidities). We hypothesize that three potential changes induced by chronic PE can occur in vitiligo patients: (i) a positive immunomodulatory response (change the balance of inflammatory to an anti-inflammatory state), (ii) improvement in EAS, and (iii) improvement in the mitochondrial structure and function (resulting in the decrease of OS). Clinically, these biochemistry/metabolic/structural changes promoted by chronic PE training can stabilize the vitiligo, prevent the spread or reappearance of vitiligo after its stabilization and potentially promote the repigmentation of affected areas. Therefore, this paper highlights the clinical applicability of a structured PE training in vitiligo disease development and proposes a new research avenue to explore the potential role of PE training in vitiligo pathophysiological mechanisms and as treatment strategy.

## Overview of Vitiligo Development

Several genetic polymorphisms have been identified in vitiligo development; such alterations come from the innate and adaptive IS and melanocytes morphology and metabolism (Bergqvist and Ezzedine, 2020).

**FIGURE 1:**
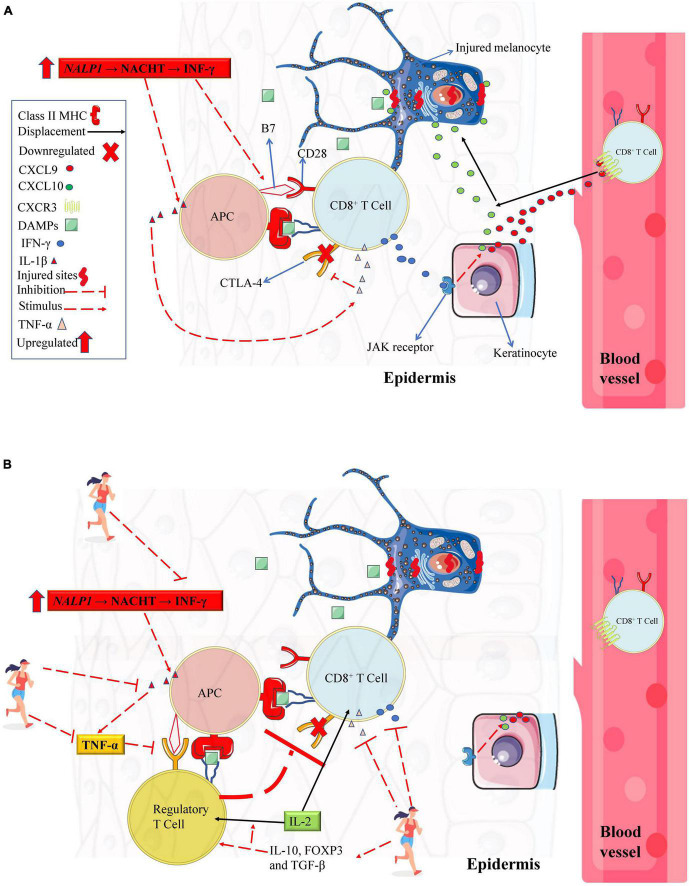
Immune system profile in melanocytes from vitiligo patients. **(A)** Vitiligo patients have genetic polymorphisms that downregulate CTLA-4 from CD8^+^ T cell and a mutation of the NALP1 gene (which results in a higher IFN-γ gene expression). These alterations might result in a greater IFN- γ gene expression upregulating the costimulatory molecule B7 from APC. Upon antigen presentation, the APC activates CD8^+^ T cell via B7-CD28 interaction (in contrast, if B7 binds to CTLA-4 receptor, the CD8^+^ T cell remains inactivated albeit antigen presentation). Upon activation, CD8^+^ T cell secrets large amounts of IFN-γ, stimulating keratinocytes (via JAK/STAT pathway) to secret chemokines (CXCL9 and CXCL10) that recruit recirculation effector CD8^+^ T cell in injured melanocytes inducing its apoptosis. In addition, activated CD8^+^ T cells secrets large amounts of TNF-α, resulting in further downregulation in CTLA-4 receptors. **(B)** A theoretical model describing the potential role of chronic physical exercise on cytotoxic T cells suppressing. Chronic physical exercise (PE) decreases pro-inflammatory cytokines such as IL-β, IFN-γ, and TNF-α and increases anti-inflammatory IL-10. Inhibiting IL-1-β and TNF-α, there are no negative feedback for CTL-4 expression in cytotoxic T cell or regulatory T cell. When the CTL-4 receptor interacts with costimulatory molecule B7 from APC (because costimulatory molecule B7 has a greater affinity for CTLA-4 than the CD28 receptor), the regulatory T cell suppresses CD8^+^ T cell activation. Also, due to chronic PE, the increase in IL-10, FOXP3, and TGF-β can stimulate regulatory T cells’ transcription, differentiation, and proliferation. An increase in regulatory T cells suppresses autoreactive cytotoxic T cells responses. The increase in regulatory T cells can consume IL-2, which is important for maintaining memory T cells, thus potentiating the decrease in the excessive memory T cell as discussed in the paper. APC, antigen-presenting cell; CD28, Cluster of Differentiation 28; CTLA-4, cytotoxic T lymphocyte antigen-4; CXCL, C-X-C motif chemokine ligand; CXCR, C-X-C motif chemokine receptor; DAMPs, damage-associated molecular pattern; JAK/STAT, Janus kinase/signal transducer and activator of transcription; IFN-γ, interferon-gamma; IL, interleukin; MHC, major histocompatibility complex; TGF-β, transforming growth factor, TNF-α, tumor necrosis factor-alpha. Figure created with images from smart.servier.com and storyset.com.

In 2007, alterations in the NALP1 genomic region were identified in vitiligo patients (Jin et al., 2007). NALP1 encodes the NACHT Leucine-rich repeat protein 1 (a cytosolic pattern recognition receptors, which are highly expressed in T cells and Langerhans cells) that detect infection or cell damage in the cytosol (e.g., DAMPs). NACHT Leucine-rich repeat protein 1 recognize pathogen-associated molecular patterns and DAMPs and recruit other proteins to form signaling complexes that promote inflammation or type I interferon production (Jin et al., 2007; Abbas et al., 2019b). Posteriorly, an upregulation in interferon-gamma (IFN-γ) gene expression has been identified in the serum from vitiligo patients (Dwivedi et al., 2013). Further, low expression of CTLA-4 (cytotoxic T lymphocyte antigen-4) in T cells was also associated with higher vitiligo disease susceptibility (Ni et al., 2014). It is well established that NACHT actively promotes IL-1β and IFN-γ gene expression. IFN-γ, in turn, induces B7 gene expression in antigen-presenting cells (APC) in the epidermis (also known as Langerhans’ cells) (Deng et al., 2018). Thus, low CTLA-4 expression in cytotoxic T cells (Song et al., 2013) increases the incidence of APC antigen presentation and cytotoxic T cells activation via B7 (from APC) to CD28 cytotoxic T cell binding (Abbas et al., 2019a, see Figure 1). Consequently, when this immunometabolism occurs with antigen from melanocytes, immune self-tolerance is lost, and melanocytes apoptosis occurs via accessory pathway activation. In contrast, in a vitiligo mouse model, it was demonstrated that an increase in regulatory T cells (Tregs) suppresses autoreactive cytotoxic T cells responses (Le Poole and Mehrotra, 2017). However, compared to healthy peers, vitiligo patients have low Tregs gene expression (and CTLA-4, as previously mentioned), which has a significant role in vitiligo development (Giri et al., 2020). Future studies are needed to explore a better strategy to promote increases in Tregs from vitiligo patients as illustrated in Figure 1B.

**FIGURE 2:**
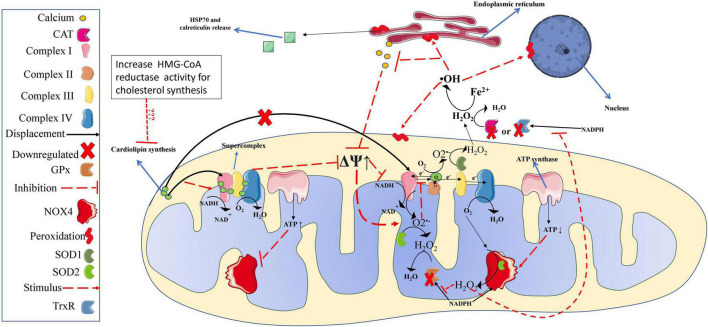
Redox balance in melanocytes from vitiligo patients. Mitochondria of melanocytes from vitiligo patients have significant defects in complex I, which generate high amounts of ROS. Lower production and distribution of cardiolipin, which plays an essential role in complex I (and supercomplexes) structure and function, are responsible for the high mitochondrial superoxide production. However, superoxide production is efficiently amortized by SOD activity, converting them into hydrogen peroxide. In Vitiligo patients, NOX4 produces significant amounts of hydrogen peroxide (from oxygen); however, the higher NOX4 activity consumes NADPH molecules used as CAT, GPx, and TrxR cofactors in the hydrogen peroxide buffer activity. Also, hydrogen peroxide excess impairs CAT, GPx, and TrxR buffer activity. Thus, the hydrogen peroxide can reach critical values undergoing significant Fento reactions producing large amounts of hydroxyl radical, leading proteins, membranes (mitochondrial and cellular), and DNA peroxidation (oxidative stress). In melanocytes from vitiligo patients, the oxidative stress leads to a loss of calcium metabolism, which will imply a worsening in the mitochondrial structuring and function (i.e., further increase the ROS production associated with lower ATP levels). Such conditions lead melanocyte organelles to significant damage and release calreticulin and HSP70, acting as DAMPs and activating the immune response described in Figure 1 (references are provided throughout the text). OH, hydroxyl radical; mitochondrial membrane potential; ATP, adenosine triphosphate; CAT, catalase; DAMPs, damage-associated molecular patterns; GPx, glutathione peroxidase, Fe^2+^, iron ferrous; H_2_O, water; H_2_O_2_, hydrogen peroxide; HSP70, Heat shock protein 70; NAD^+^, oxidized form of nicotinamide adenine dinucleotide; NADH, reduced form of nicotinamide adenine dinucleotide; NADPH, reduced form of nicotinamide adenine dinucleotide phosphate; NOX2, NADPH oxidase isoform 2; NOX4, NADPH oxidase isoform 4; O2^–^, superoxide, PE, physical exercise; ROS, reactive oxygen species; SOD, superoxide dismutase; TrxR, thioredoxin reductase.

In vitiligo patients, high IFN-γ expression has been implicated in an aggressive and permanent IS response (IFN-γ→ CXCL10 (C-X-C Motif Chemokine Ligand 10) → CD8^+^ T cells) (Xie et al., 2016) targeting the population of epithelial cells throughout the body causing visual (Agarwala and Malkud, 2020), hearing (Ma et al., 2021) and vascular dysfunction (Azzazi et al., 2021). Nonetheless, one of the main visible features of this clinical condition is noted when the IS attacks epidermal melanocytes (Dwivedi et al., 2013), leading them to apoptosis without replacement, a phenotypic trait of vitiligo, identified by white spots in the skin, which signalize the absence of melanocytes at the site (Rashighi et al., 2014). The mechanistic trigger for loss of immune self-tolerance (which induces melanocytes apoptosis) is dysfunctional mitochondria resident in epidermal melanocytes (Dell’Anna et al., 2007) and also in CD8^+^ T cells (Dell’Anna et al., 2003). These defective mitochondria have low concentration and abnormal cardiolipin distribution in the mitochondrial electron transport chain (mETC), which cause defects in complex I formation, impairing the stability and creation of mitochondria supercomplexes (essential for normal ATP production and low mitochondrial ROS emission (see scheme in Figure 2; Dell’Anna et al., 2003, 2007; Dell’Anna et al., 2017). Interestingly, in an *in vitro* study, cardiolipin replacement rescued normal mitochondria function from vitiligo patients (Dell’Anna et al., 2017). However, more studies are needed to determine the cause of low cardiolipin concentration in mitochondria from melanocytes and CD8^+^ T cells and how to restore the normal concentrations of this lipid *in vivo*.

In addition to high mitochondrial ROS emission, vitiligo patients have a deficient EAS, with low activity and low gene expression of catalase (CAT), glutathione peroxidase (GPx), and thioredoxin reductase (TrxR) (Laddha et al., 2013; Xie et al., 2016), leading to a chronic OS state (Schallreuter, 2014). The low EAS effectiveness in vitiligo patients is related to its disabling caused by the chronic OS (Schallreuter et al., 1991a; Schallreuter, 2014). High OS causes cell damage leading melanocytes to secrete autoantigens such as calreticulin and heat shock protein 70 (Hsp70) acting as DAMPs (Mosenson, 2013; Zhang et al., 2014; Xie et al., 2016), which induces an adaptive immune response, via cytokines such as interleukin (IL)-1β, IL-6, IL-12, interferon (IFN)-α, IFN-γ, and tumor necrosis factor-alpha (TNF-α) (Asea, 2007; Xie et al., 2016).

### A Possible Link Between Vitiligo Onset and Progression With Sedentary Lifestyle

As with other diseases, patients’ environmental context modulates their genetic predisposition. Despite the consensus on the role of genetic predisposition and the greater susceptibility of vitiligo development, in monozygotic twins, vitiligo develops in only 23% of both twins (Alkhateeb et al., 2003), suggesting that the environment can induce or suppress the genes related to vitiligo development via DNA methylation, histone modifications and alteration of circulating microRNA (Zhou et al., 2019). For example, it has been identified that there is global hypermethylation of DNA in PBMCs, particularly in regions related to the increase of IL-10 in vitiligo patients (Zhao et al., 2010). Also, when compared to their healthy peers, vitiligo patients have a high serological concentration (Shi et al., 2016) and different miRNA profile expression in PBMC (Shi et al., 2013) that can characterize this population. The vitiligo miRNAs profile is related to melanocyte metabolism (Shi et al., 2016) and immune system regulation (Wang et al., 2015), such as cytokine profile to CD8^+^ T cell upregulation in PBMCs (Zhou et al., 2019; Zhang et al., 2021) and melanocytes degeneration (Wang et al., 2015). It is well known that chronic PE also imposes strong epigenetic alteration on the immune system (Antrobus et al., 2021), EAS enzymes (Dimauro et al., 2020), and mitochondrial structure and function (Pareja-Galeano et al., 2014) throughout DNA methylation, post-translational histone modification, and microRNA transcripts. For example, data from the literature demonstrate that acute exercise induces PBMCs hypomethylation (Horsburgh et al., 2015), which might upregulate IL-10 expression in vitiligo patients (Zhao et al., 2010). In fact, vitiligo development has an epigenetic background that leads to its development, and it is plausible that chronic PE can recover it. Therefore, future studies deepening discussing this topic (if chronic PE is capable of rescuing the healthy epigenetic profile of vitiligo patients) is guaranteed and highly needed.

Today, little attention has been paid to the role of environmental factors like diet or PE training or the level of habitual physical activity in vitiligo disease. In fact, to the best of our knowledge, there are no studies focused on PE or habitual physical activity level and vitiligo disease, although several studies with similar etiological factors have consistently identified significant associations between vitiligo (as well as its severity) with MetS (Ataş and Gönül, 2017; Sharma et al., 2017; Tanacan and Atakan, 2020; Verma et al., 2021) or related dysfunctions such as higher blood plasma concentrations of low-density lipoprotein (LDL- cholesterol), low high-density lipoprotein (HDL-cholesterol), and insulin resistance (Karadag et al., 2011; Azzazi et al., 2021; Demirbaş et al., 2021; D’Arino et al., 2021). It is well established that a mainly sedentary lifestyle behavior and poor nutritional patterns are the most important risk factors for the onset of MetS or cholesterol disorders (Lira et al., 2010; Edwardson et al., 2012). Further, OS and the predominance of pro-inflammatory cytokines are also established mechanistic factors involved in MetS development (Bonomini et al., 2015). There is no study identifying a cause/effect relationship between MetS and vitiligo although vitiligo severity is associated with MetS. However, the incidence and prevalence of MetS or associated risk factors such as insulin resistance, hypertension, LDL-cholesterol, obesity, or large abdominal circumference are higher in vitiligo patients (Ataş and Gönül, 2017; Sharma et al., 2017; Tanacan and Atakan, 2020; Namazi et al., 2021). It is plausible that excessive ROS from other metabolic disorders (such as obesity, insulin resistance, and elevated LDL-cholesterol and low HDL-cholesterol) can induce a state of chronic low-grade inflammation (e.g., raising IFN-γ and TNF-α), possibly aggravating or activating vitiligo in genetically predisposed individuals, as illustrated in Figure 1. As discussed, and partially supporting this rationale, high levels of OS and IFN-γ are the mechanistic trigger for vitiligo manifestation (Laddha et al., 2014; Xie et al., 2016). Also, data from vitiligo patients in Dell’Anna et al. (2007, 2010), *in vitro* mechanistic study (Hauff et al., 2009), and animal model (Han et al., 2007) indicated that higher demand for cholesterol synthesis (e.g., for cell grow or LDL-cholesterol increases) is detrimental to mitochondrial cardiolipin content. This rationale is supported by data who statin mitigating the vitiligo spreading (Hasan et al., 2021). Statins have a lower effect on LDL-cholesterol via HMG-CoA reductase inhibition. HMG-CoA reductase is the rate limit for cholesterol synthesis and competes with cardiolipin for the cardiolipin synthase function (Hauff et al., 2009). Therefore, a link between lifestyle and vitiligo exists, and future experimental studies are needed to verify these hypotheses.

In vitiligo patients, these three factors (hyper-reactive IS, chronic OS, and deficient EAS) interact in an interdependent and reciprocal way. For instance, the increase in OS through an augment in defective mitochondria and an inefficient EAS trigger and maintain a specific and chronic inflammatory response in vitiligo patients (via IFN-y → CXCL10 → CD8^+^ T cells) (Laddha et al., 2013; Xie et al., 2016). The increase of this pro-inflammatory state, in turn, also maintains OS and disables the EAS in a loop process that will remain active indefinitely, inducing apoptosis of melanocytes without its proper replacement leading to vitiligo scars and disease progression through time (Ortona et al., 2014; Lotti et al., 2015; Xie et al., 2016). Along with this metabolic profile in vitiligo patients, a lifestyle whose etiological factors are also based on low-chronic inflammation, OS, and inefficient EAS lead to the development of disorders within MetS scope and worsen vitiligo’s condition.

To stop vitiligo progression and promote a favorable environment for repigmentation (maturation of melanocytes), is necessary an intervention that reduces ROS production, improves the EAS, and reduces the pro-inflammatory profile (Lotti et al., 2015). The use of antioxidants or immunosuppressant drugs to reduce ROS production is already widely used in clinical practice (Bergqvist and Ezzedine, 2020). However, these therapeutic strategies do not treat the source of ROS, being a temporary solution that does not prevent vitiligo reappearance. Further, there is no proposed intervention to improve EAS in vitiligo patients. In item 2.2, we will discuss how OS is established in vitiligo patients and how they trigger the IS response to melanocytes. We will use this information to suggest the structured physical training program (item 3) to reduce the OS condition, improve EAS, and modulate the IS.

### Origin of Oxidative Stress and Vulnerable Antioxidant System: Mechanistic Factors in Vitiligo Development

It is well known in vitiligo patients that dramatic imbalance between the oxidant system (high) and EAS (deficient) leads to a predominant state of OS (Schallreuter, 2014). A higher rate of lipid peroxidation (LP) exists in individuals with vitiligo compared to healthy counterparts or generalized vitiligo compared to individuals with localized vitiligo; also, individuals with active vitiligo compared to individuals with stable vitiligo has a higher LP (Laddha et al., 2013, 2014). Thus, a greater OS results in more aggressive and severe vitiligo disease for these patients. As presented in Figure 2, the magnitude of ROS production in melanocytes in vitiligo patients is due to a high electrical potential in mitochondrial intermembrane space (Dell’Anna et al., 2017). These individuals have an altered mETC with a reduced distribution of cardiolipin, which leads to a defective complex I formation (Dell’Anna et al., 2017). Cardiolipins are responsible for configuring and forming mETC complexes and supercomplexes assembly (Schlame and Greenberg, 2017). Complex I is the rate-limiting step of aerobic respiration and has a central role in energy production for metabolism (Sharma et al., 2009). Therefore, disturbance in cardiolipin concentrations or distribution will lead mitochondria to inefficient energy production.

Dell’Anna et al. (2007) showed that cardiolipin concentration and distribution disturbances lead to inefficient mitochondrial melanocytes’ ATP production, low glucose consumption, and high ROS emission (Dell’Anna et al., 2007). Recently, another research group (Martins et al., 2021) also showed that vitiligo skin T cells induce melanocytes to produce higher oxygen consumption and ROS emission without increasing the glycolysis flux. In their experiments, the melanocytes’ ROS production was blunted when N-acetylcysteine was used as an antioxidant in the cell culture. More importantly, the excess of oxygen consumption and ROS production was blunted when ruxolitinib, a Janus kinase (jak)1/2 inhibitor, was administered in the cell culture. Taken together, these data indicated that vitiligo skin T cells induce melanocytes to increase oxygen consumption to produce ROS, overwhelming its antioxidant system. Interestingly, the lack of change in glycolysis flux in melanocytes interaction with vitiligo skin T cells (via jak/STAT signaling described in Figure 1A) suggests that ROS production (with the extra oxygen consumption) over to ATP production (as illustrated in Figure 2), as discussed in this paper, is a profile to activating cell apoptosis in epidermal from vitiligo patients.

The major ROS product found in vitiligo patients is hydrogen peroxide (H_2_O_2_) which plays a crucial role in LP and in disease development (Schallreuter, 2014). The enzymatic complex superoxide dismutase (SOD) and Reduced nicotinamide adenine dinucleotide phosphate- oxidase (NADPH-oxidase, specifically, the NOX4 isoform localized in the mitochondria) have been identified as the main source of H_2_O_2_ (Laddha et al., 2013; Barygina et al., 2015). The SOD complex has a high gene expression and enzymatic activity in individuals with active vitiligo (Glassman, 2014), probably due to elevated ROS emission from defective mitochondria (Dell’Anna et al., 2010). Elevated H_2_O_2_ production by SOD activity leads to an early inhibition of EAS enzymes activity such as TrxR (Schallreuter et al., 1991a), CAT and GPx, which in turn, leads to an increase in Fenton reaction, increasing hydroxyl concentrations (Fe^2+^ + H_2_O_2_ → Fe^3+^ + OH^–^ + •OH) to a toxic level, that ultimately, leads to a significant peroxidation in several cellular components (membranes, proteins, mitochondria, and DNA) (Schallreuter, 2014). To remove H_2_O_2_ excess, GPx, TrxR, and CAT (i.e., enzymes involved in EAS) are dependent on reduced nicotinamide adenine dinucleotide phosphate (NAPDH) for the renewal of its substrate, thereby enabling the clearance of ROS. However, the NADPH-oxidase activity (which also uses NADPH as a cofactor) is increased (producing H_2_O_2_) during active vitiligo, therefore increasing ROS and promoting an active competition with EAS enzymes for NADPH molecules, a molecule already reduced in active vitiligo patients (Shajil and Begum, 2006). Thus, with the significant increases in H_2_O_2_ concentrations, the NADPH binding site is inhibited with EAS enzymes (Schallreuter, 2014). Therefore, the EAS enzymes are crucial for preventing systemic OS, i.e., containing H_2_O_2_ extravasation from the mitochondria and their respective melanocyte cells to the other sites (Lu and Holmgren, 2014); however, EAS enzymes are deactivated due to chronic elevated H_2_O_2_ induced by defective mitochondria (Schallreuter, 2014). Together, these studies demonstrate that the removal of ROS in vitiligo patients is a deficient process.

The increase in H_2_O_2_ drastically impairs calcium metabolism in the epidermis (both cell influx and efflux, as well as the structure of L-type calcium channels) (Schallreuter, 2014). This process also causes inhibition in the ROS removal processes, regulation of melanin biosynthesis, and DNA repair via allosteric regulation of TrxR by calcium metabolism (Schallreuter et al., 1991a; Gafter et al., 1997; Schallreuter, 2014). The TrxR proper functions are calcium-dependent (TrxR + Ca^2+^ = TrxS_2_ + NADPH Trx(SH)_2_ + NADP^+^); thus, both pigmentation and ROS removal may be compromised if the melanocytes do not have a stable calcium metabolism (Schallreuter and Wood, 1991). It has been demonstrated that the areas of the epidermis affected by vitiligo have a poor calcium metabolism, with low calcium uptake by melanocytes or an absence of L-type calcium channels (Schallreuter-Wood et al., 1996; Schallreuter et al., 2012). With dysfunctional calcium metabolism, patients with vitiligo also have low melatonin, serotonin (consequently, increased tryptophan), and high noradrenaline concentrations in the epidermis, which further increases the OS (Schallreuter, 2014).

**FIGURE 3:**
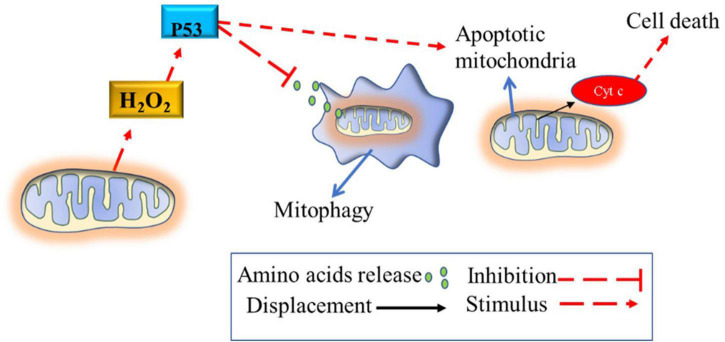
Blunted mitophagy in melanocytes of vitiligo patients. The mitophagy process of defective mitochondria is inhibited by the high activity of P53, which is stimulated by the high production of hydrogen peroxide. Thus, defective mitochondria continue to produce reactive oxygen species, causing oxidative stress until the cellular apoptosis process is induced via mitochondrial cytochrome c release or via the cytotoxic T cell pathway recruitment (described in Figure 1A). The process of mitophagy destroys defective mitochondria releasing amino acids into the cellular cytosol that can be used for other cellular functions. H_2_O_2_, hydrogen peroxide; p53, tumor suppressor p53.

The absence of calcium in the mitochondria due to chronic higher H_2_O_2_ production causes mitochondrial swelling (Schallreuter, 2014; Tsai et al., 2014; Ding et al., 2015). Notably, melanocytes from vitiligo patients are known to have altered mitochondria morphology that does not undergo the mitophagy process. These mitochondria are swollen with obscure and vacuolated ridges, especially in individuals with active vitiligo (Ding et al., 2015). These mitochondrial morphological changes may be related to high gene expression and the ability to stimulate the activity of the P53 tumor suppressor protein (Teulings et al., 2013) and SOD enzymes (Laddha et al., 2013); and also to suppress the mitophagy process in this pathology (Lionaki et al., 2015). The lack of melanocytes’ mitophagy process suggests that highly damaged mitochondria continue to produce high amounts of ROS, which will lead cell to apoptosis (Figure 3). Collectively, these studies suggest that chronic high H_2_O_2_ concentration, *per se*, disable EAS enzymes, calcium metabolism and inhibits the mitophagy process in melanocytes; in turn, these mechanisms lead to melanocytes destruction (via IS stimulation) and to the impossibility of their maturation and resynthesis.

In summary, this evidence suggests that the origin of excess ROS production is the result of (1) defective mitochondria (due to the abnormal and reduced distribution of cardiolipins) with high intermembrane electrical potential and (2) reduced mitophagy process (due to high expression and activity of P53 protein). Furthermore, there appears to be a vulnerability or depletion of EAS enzymes to limit the excess ROS produced by the defective’s mitochondria.

#### Reciprocal Action of Oxidative Stress and Immune System in Vitiligo

Laddha et al. (2014) verified for the first time the relationship between OS and IS in the development of vitiligo and found that melanocytes ROS overproduction precedes the immune response. Vitiligo melanocytes’ have a vulnerable EAS, as previously discussed, its chronic exposure to OS promotes the increase of several autoantigen markers (DAMPs), such as the exposure of calreticulin on the cell surface and the secretion of Hsp70 to the extracellular matrix triggering a IS response (Zhang et al., 2014; Xie et al., 2016). An animal model study has been shown that Hsp70 secretion by melanocytes induces the progression of vitiligo (Mosenson, 2013). Extracellular Hsp70 is a potent inducer of the innate and adaptive immune response. For instance, it induces the release of cytokines such as TNF-α, IL-1β, IL-6, and IL-12. In contrast, intracellular Hsp70 has a cytoprotective, anti-apoptotic, and anti-inflammatory role (Asea, 2007). Situations of psychological stress or trauma (cellular damage), pre-apoptotic cells (necrosis) secrete high concentrations of IFN-γ and ROS, which stimulates Hsp70 released to the extracellular environment (Asea, 2007). This rationale is partially supported by the fact that individuals with vitiligo in the active phase develop depigmentation in areas that suffer mechanical trauma (Lee et al., 2004).

A case study showed that the increase in IFN-γ (as a form of treatment for other pathologies) caused the appearance of vitiligo (Kocer et al., 2009), and treatment with anti-IFN-γ significantly decreased vitiligo progression (Skurkovich and Skurkovich, 2006). Both in humans and in rats, IFN-γ modulates the pigmentation state of melanocytes, but in high concentrations limits melanocyte’s maturation and differentiation (Natarajan et al., 2014). Dwivedi et al. (2013) showed that in patients with active vitiligo (appearance of recent spots), there is a higher IFN-γ gene expression than in individuals without vitiligo or with stable vitiligo (individuals without the occurrence of new spots in the last six months). Furthermore, in animal models (rats) and in perilesional skin from vitiligo patients, the increase in IFN-γ, IFN-α, TNF-α, cytotoxic granulation marker (CD107a) (You et al., 2013; Bertolotti et al., 2014) were positively correlated with CD8^+^ T cells activation in melanocytes and with vitiligo severity (melanocytes destruction without their replacement). This evidence suggests a feedback loop between innate and adaptative immune responses in melanocyte’s skin in active vitiligo. As previously referred, this loop is fed by melanocytes OS, which leads melanocytes to secret DAMPs and stimulating IFN-γ production, a pivotal cytokine to recruit and guide recirculating CD8^+^ T cells to melanocytes via IFN-γ–CXCL9/10–CXCR3 axis (Figure 1).

Elevated concentrations of specific melanocytes CD8^+^ memory T cells (secreting high IFN-γ) were found in vitiligo patients, mainly during active disease state (Riding and Harris, 2019). The maintenance of memory CD8^+^ T cells in depigmented areas prevents repigmentation and is responsible for the disease recurrence after treatment interruption. It has been described that the progression and recurrence of vitiligo occur when memory CD8^+^ T cells residing in the epidermis secrete IFN- γ (in response to DAMPs from melanocytes), which induces the recruitment of recirculating memory CD8^+^ T cells (Riding and Harris, 2019). It is important to refer that this mechanism of melanocytes apoptosis seems to occur in a low rate of Tregs/CD8^+^ T cells, i.e., insufficient Tregs suppressing melanocyte-specific CD8^+^ T cells, putting in anergy state or suppressing (Riding and Harris, 2019). It has been suggested that successful treatments need to neutralize the activity of recirculating memory CD8^+^ T cells or memory cells residing in melanocytes (e.g., by immunosuppression) to prevent disease progression (Rashighi et al., 2014; Riding and Harris, 2019).

Vitiligo patients can be characterized by a pro-inflammatory profile with high expression of T helper (Th) 1 and Th17 populations and low expression of Treg and Th2 populations. The literature reports a higher concentration of inflammatory cytokines markers such as IL-2, IL-6, IL-15, TNF-α, and IFN- γ and, in contrast, decreased anti-inflammatory cytokines like IL-4 and IL-10 (Lotti et al., 2015; Riding and Harris, 2019).

Overall, continued OS production can sustain chronic inflammation indefinitely (Lotti et al., 2015). This chronic inflammation plays a key role in the repigmentation process of vitiligo wounds (Lotti et al., 2015). It has been long known that the repigmentation process of the affected areas needs the migration and proliferation of immature melanocytes as well as the reestablishment of calcium metabolism. However, these cell renewal processes do not occur when high levels of pro-inflammatory cytokines and/or OS are predominant in depigmented areas (Schallreuter, 2014). Taken together, an effective treatment to limit vitiligo progression and recurrence needs to improve i) the EAS enzymes (i.e., GPx, TrxR, and CAT); ii) the function and quality of mitochondria structure in melanocytes and CD8^+^ T cells; and iii) change the profile of the immune system.

## Potential Role of Chronic Physical Exercise on Vitiligo Development

Chronic PE has great potential to improve EAS (consequently decreasing OS), mitochondrial function and decrease the inflammatory profile (Table 1). Hypothetically, this modulation can prevent vitiligo progression and probably facilitate vitiligo wound healing. Nonetheless, to date, there are no studies analyzing the clinical applicability of PE training or physical activity level in vitiligo disease. Therefore, based on state-of-the-art evidence from PE literature (redox system, mitochondrial function/structure, and immunology), we will propose *PE* as a potential therapeutic strategy on vitiligo disease as summarized in Table 1.

**TABLE 1:** The potential role of physical exercise training on vitiligo patients.

Modifiable vitiligo profile	Potential from acute exercise	Potential from physical training
**Metabolic profile**		
It is associated to MetS and ↑ insulin resistance, adipose tissue, blood pressure, LDL-C, and ↓ HDL-C.	↑ gene transcription to glucose uptake, fat oxidation, cardiac and vascular remodeling.	↓ insulin resistance. ↓ LDL-C and blood pressure. ↑HDL-C. Improvement in several MetS markers. ↓ adipose tissue.
**Redox system profile**		
↑ ROS/RNS. ↓ EAS enzyme (GPx, TrxR, and CAT). Chronic ↑ NADPH-oxidase and SOD activity. ↑Lipid, protein, and DNA peroxidation.	Acute ↑ NADPH-oxidase activity induces EAS enzymes gene transcription (via NF-κB pathway). ↑ Nrf2-ARE/HO-1 pathway activation.	↑ EAS enzymes capacity and ↓ lipid, protein, and DNA peroxidation. ↓ROS. ↓ NADPH-oxidase activity-induced ROS. ↑ NADPH synthesis.
**Mitochondrial structure and function profile**		
↓ mitochondrial mitophagy in melanocytes. ↓ cardiolipin quality and quantity; ↓ melanocytes mitochondrial ATP production and mitochondrial complexes and supercomplex activity. ↑ mitochondrial ROS emission.	↑ gene transcription for mitochondrial biogenesis and remodeling. ↑ IGF-1/PI3K/AKT/ACL pathway activation to cardiolipin biosynthesis.	↑ mitochondrial mitophagy and remodeling (mitofision, mitofusion). ↑ ATP mitochondrial (increase in complex 1) and mitochondrial mass. ↑ cardiolipin content and supercomplex formation and ATP content. ↓ mitochondrial ROS emission.
**Immune function profile**		
↑ IL-2, IL-6, and IL-15. ↓ IL-4 and IL-10. ↑ TNF-α, IFNα, and IFN- γ. ↑ memory CD8^+^ T cell. ↓Tregs. ↑ extracellular HSP.	CD8^+^ T cells mobilization to bloodstream removing hyper-reactive senescent cells. ↑ acute increase in IFN-α, IL-6, and IL-1β inducing a regulatory effect in IL-4, IL-10, and IL-1RA.	↓ IL-2, IL-6, and ↑ IL-15 ↑ IL-10 and IL-4. ↓ TNF-α and IFN- γ. ↓ total lymphocytes, CD8^+^ T cell proliferation, memory CD8^+^ T cell, and ↑ senescent CD8^+^ T cell apoptosis. ↑ Tregs. ↓ extracellular HSP and ↑ intracellular HSP

*References from this table are provided as Supplementary Material. ↑, increase; ↓, decrease; ATP, adenosine triphosphate; CAT, catalase; EAS, endogenous antioxidant system; GPx, glutathione peroxidase; HSP, heat shock protein; HDL-C, high-density lipoprotein- cholesterol; HSP, heat shock protein; IFN, interferon; IGF-1, insulin-like growth factor 1; IL, interleukin; LP, lipid peroxidation; LDL-C, low-density lipoprotein- cholesterol; MetS, metabolic syndrome; Nrf2-ARE/HO-1, Nuclear Factor E2 related to Factor 2-antioxidant/heme oxygenase 1 response element; ROS/RNS, reactive oxygen species/reactive nitrogen species; SOD, superoxide dismutase; TNF, tumor necrose factor; Tregs, regulatory T cells; TrxR, thioredoxin reductase.*

### Potential Role of Chronic Physical Exercise on Redox System Modulation: Implications on Vitiligo Development

Vitiligo Patients have an associated high rate of inflammatory comorbidities such as MetS and cardiovascular diseases (Lotti and D’Erme, 2014). These diseases are also etiologically related to a higher OS. Notably, accumulating evidence has shown that structured chronic PE training or high cardiorespiratory fitness (CRF) decreases the risk of OS-related pathologies (Farrell et al., 2012; de Sousa et al., 2016; Lu et al., 2021).

Initially, acute PE (mainly the aerobic-type) was believed to be a bad approach once it increases 20 times oxygen consumption and sharply increases ROS and reactive nitrogen species (RNS) production (Leeuwenburgh and Heinecke, 2001). Paradoxically, chronic PE was known to induce a positive decrease in ROS production during acute exercise and at rest (Leeuwenburgh and Heinecke, 2001). Today, it is well established that skeletal muscle ROS produced during acute PE is responsible for several positive adaptations in skeletal muscle and other tissues. Each bout of PE (resistance or aerobic-type) induces putative ROS/RNS homeostasis, which is necessary to induce gene translation related to aerobic metabolism (e.g., mitochondrial proteins) and to muscle protein synthesis (e.g., EAS enzymes) (Carlos Henríquez-Olguín et al., 2019; Vargas-Mendoza et al., 2019). Therefore, the well-established concept that individuals with high CRF have low ROS levels (when compared to individuals with low CRF) is a direct result of acute ROS stimulation by PE bouts. For instance, improved mitochondrial function due to aerobic training results in decreased mitochondrial electrical potential. In physically trained individuals, OS becomes smaller both at rest and during acute PE when compared with sedentary individuals (Venditti et al., 1999). Further, the hormetic effect of chronic PE (Radak et al., 2014; Margaritelis et al., 2018; Aguiar et al., 2021) is an efficient tool to promote improvement in EAS, especially in susceptible individuals to a greater situation of OS such as obesity, type 2 diabetes (T2D) or with cardiovascular disease (Radak et al., 2014; de Sousa et al., 2016). Even in healthy individuals, chronic PE (especially aerobic types) leads to redox system adaptations that decrease OS levels (Margaritelis et al., 2018; Aguiar et al., 2021). Finally, it is important to mention the conclusions of a meta-analysis (de Sousa et al., 2016; Aguiar et al., 2021), supporting that chronic PE tends to decrease OS markers and increase the antioxidant system, without a redox imbalance after a well-designed PE program.

An elegant study (Gifford et al., 2016) showed that individuals with high CRF (VO_2_max, ∼59 ml⋅kg^–1^⋅min^–1^) have high amounts of mitochondria (i.e., “excess”), and are far from saturating mitochondrial work capacity even in high-intensity PE. In contrast, in individuals with low CRF (VO_2_max, ∼38 ml⋅kg^–1^⋅min^–1^) easily exceeded mitochondrial work capacity. According to the authors (Gifford et al., 2016), this mitochondrial “excess” or reserve mitochondria can work as ROS removal system, thus, preventing mitochondria’s mass from reaching its maximal respiratory capacity, which can damage their structures (Sansbury et al., 2011).

Vitiligo patients have a deficient endogenous EAS with low CAT activity (Maresca et al., 1997) both in the epidermis (Schallreuter et al., 1991b) and in the blood plasma (Shajil and Begum, 2006). In addition, GPx and TrxR have low activity in vitiligo patients (Schallreuter et al., 1991b; Shajil and Begum, 2006). The nuclear factor E2 related to factor 2-antioxidant/heme oxygenase 1 response element (Nrf2-ARE/HO-1), a key metabolic pathway for genetic signaling transduction of enzymes related to the antioxidant system, is impaired in vitiligo patients (Jian et al., 2014). The upregulation of the Nrf2-ARE/HO-1 axis is necessary to improve melanocytes’ tolerance to ROS stressors (mainly from H_2_O_2_). Notably, if the Nrf2-ARE/HO-1 axis metabolic pathway is stimulated in melanocytes, the ability to remove ROS in these cells is restored (Jian et al., 2014). Further, animal studies have shown that there is an increase in Nrf2 expression, increasing the expression of antioxidant enzymes after acute (Muthusamy et al., 2012) and chronic PE (Gounder et al., 2012).

Acute PE also increases signaling pathways of the enzymatic antioxidant system by NADPH-oxidase→ NF-κB (Carlos Henríquez-Olguín et al., 2019). Although NADPH-oxidase activity during acute PE is the main source of ROS, this increase in ROS is necessary to induce GPx, CAT and SOD enzymes gene expression as well as mitochondrial biogenesis via NF-κB (Henríquez-Olguín et al., 2016). On the other hand, in animal models, chronic aerobic-exercise training reduces ROS produced by NADPH-oxidase isoforms (NOX2 and NOX4) (Qi et al., 2020). However, more studies are needed to determine if chronic PE training decreases NADPH-oxidase isoforms (i.e., NOX4) activity from immune cells infiltrated in melanocytes from vitiligo patients (Barygina et al., 2015). Moreover, as the decrease in NADPH-oxidase activity results in reduced ROS levels emission (Adams et al., 2005) more studies are also needed to verify if the decrease in ROS production induced by chronic PE has clinical significance in vitiligo patients.

**FIGURE 4:**
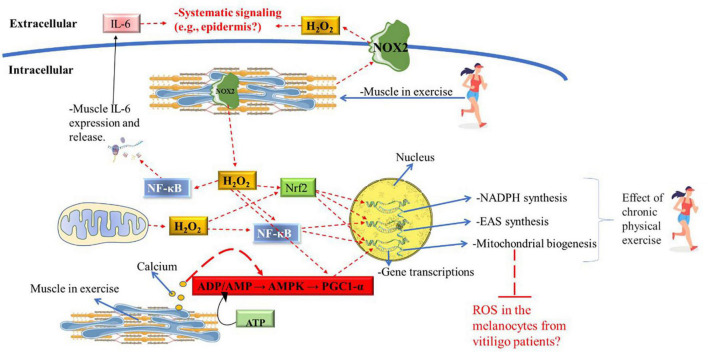
Potential role of chronic PE on redox system in vitiligo patients. Muscle contraction and mitochondrial activity produce large amounts of hydrogen peroxide during physical exercise, acting as an activation signal for the NF-κB, Nrf2, and, PGC1-α metabolic pathway downstream. ATP degradation and calcium release caused by muscle contraction also significantly induce the AMP/AMPK/PGC1-α axis. During acute exercise, gene transcription of EAS enzymes is significantly induced by the NF-κB and Nrf2o pathways; genes encode enzymes in the pentose phosphate pathway (i.e., for NADPH synthesis) is induced by the Nrf2 pathway; mitochondrial gene transcription is induced by the NF-κB, Nrf2, and PGC1-α pathways. Only chronic physical exercise will significantly induce the synthesis of mitochondria, EAS enzymes, and NADPH synthesis. Muscle contraction also releases a large amount of IL-6 and hydrogen peroxide to the extracellular medium, which may exert systemic signaling. Future research is needed to identify whether IL-6 and hydrogen peroxide from muscle tissue can exert significant signaling in the epidermis. Also, further research will be needed to assess whether the increase in mitochondria in muscle tissue can buffer the ROS produced by melanocytes in the epidermis of patients with vitiligo and whether this has clinical relevance. ADP, adenosine diphosphate; AMP, adenosine monophosphate; AMPK, adenosine monophosphate-activated protein kinase; ATP, adenosine triphosphate; H_2_O_2_, hydrogen peroxide; NF-κB, nuclear factor kappa-light-chain-enhancer of activated B cells; Nrf2, Nuclear factor-erythroid factor 2-related factor 2; IL-6, interleukin-6; NOX2, NADPH oxidase isoform 2; PE, physical exercise; ROS, reactive oxygen species.

Another mechanism by which chronic PE can improve ROS production in vitiligo disease is the increase of NADPH availability (a molecule with reduced concentrations in vitiligo patients) (Shajil and Begum, 2006). The decrease in NADPH-oxidase activity in response to chronic PE can increase the availability of NADPH for glutathione reductase to renew GSH and TRx (SH)2 (Jenkins and Goldfarb, 1993; Pannala and Dash, 2014). So, the H_2_O_2_ produced by SOD (an enzyme with high activity in vitiligo) can be properly removed by GPx and TrxR (Leeuwenburgh and Heinecke, 2001; Pannala and Dash, 2014). A schematic overview of potential role of PE on redox system modulation is illustrated in Figure 4.

### Potential Role of Chronic Physical Exercise on Immune System Modulation: Implications on Vitiligo Development

It has been well established that structured chronic PE has an anti-inflammatory effect (Gjevestad et al., 2015), especially in MetS (Alizaei Yousefabadi et al., 2020). A recent meta-analysis shows a decrease in pro-inflammatory cytokines such as IFN-γ, TNF-α, and IL-8 and increase in anti-inflammatory IL-10 after chronic PE (Alizaei Yousefabadi et al., 2020). In addition, an early systematic review pointed that chronic PE induces a decrease in Th1 cell lineage gene expression (Gjevestad et al., 2015). The Th1 gene expression is responsible for the production of IFN- γ, which induces the activation of a set of chemokines, including CXCL10, that in vitiligo patients is responsible for the chemotaxis of CD8^+^ T cells to melanocytes and to start the process of destruction of these cells (Rashighi et al., 2014). On the other hand, it is well established the anti-inflammatory role of IL-10, which induces up-regulation of Th2 that, in turn, inhibits Th1 cytokines (for example, increases Tregs cells and downregulates IFN-γ). IL-10 also inhibits antigen presentation by APC (Abbas et al., 2019a; see Figure 1B).

**FIGURE 5:**
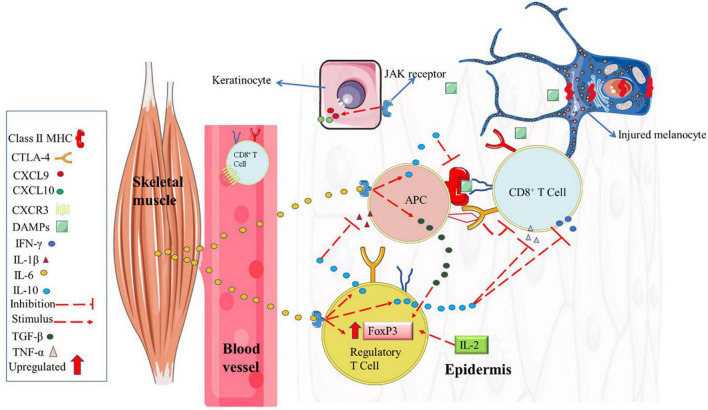
Potential role of chronic physical exercise on immune system modulation in vitiligo patients. Physical exercise can induce skeletal muscle to release huge amounts of IL-6, which has the potential to reach the skin and induce a local adaptation of the immune system (anti-inflammatory profile). For example, hypothetically high amounts of IL-6 in the skin can be detected by skin-resident APC and regulatory T cells that, in turn, give negative feedback releasing IL-10. IL-10 can inhibit the local production of IL-1, IFN-γ, and TNF-α. Inhibiting IL-1-β and TNF-α, there is no negative feedback for CTLA-4 expression in cytotoxic T cells (the interaction between CTL-4 from CD8^+^ T cell with costimulatory molecule B7 from APC suppress CD8^+^ T cell activation). Also, IL-10 could inhibit CD8^+^ T cells by inhibiting the expression of costimulatory class II MHC molecules from APC. IL-6 also induces significant TGF-β expression, resulting in upregulation of FoxP3. APC, antigen-presenting cell; CD28, Cluster of Differentiation 28; CTLA-4, cytotoxic T lymphocyte antigen-4; CXCL, C-X-C motif chemokine ligand; CXCR, C-X-C motif chemokine receptor; DAMPs, damage-associated molecular pattern; JAK/STAT, Janus kinase/signal transducer and activator of transcription; IFN-γ, interferon-gamma; IL, interleukin; MHC, major histocompatibility complex; TGF-β, transforming growth factor, TNF-α, tumor necrosis factor-alpha. Figure created with images from smart.servier.com.

It is well known that the main anti-inflammatory effect from chronic PE derives from the acute large release of muscle IL-6 (during acute exercise bouts) to the bloodstream, which exerts a paracrine effect in several tissues and organs such as the brain, bones, and digestive tract (Ellingsgaard et al., 2019). IL-6 from muscle tissues stimulated by acute PE modulates the Treg and Th17 cells ratio via IL-10 (Figure 5). This process decreases IS autoreactive potential by increasing Treg and decreasing Th17 ratio, which is a process opposite to vitiligo onset (Lotti et al., 2015; Xie et al., 2016). Also, it appears that muscle release of IL-6 plays an important role in the PBMCs hypomethylation (Horsburgh et al., 2015).

The decrease in Th1 and Th17 expression, and the increase in Treg and Th2 population, is also related to a powerful effect that chronic PE exerts on Hsp70. It was recently shown that acute PE increases extracellular Hsp70 concentrations (more specifically in blood plasma); however, it was followed by a concomitant increase in intracellular Hsp70 concentration (more specifically in peripheral blood mononuclear cells- PBMCs) (Lee et al., 2015). As a chronic effect of PE training on Hsp70 in PBMCs, there is an increase in intracellular at basal levels associated with their decrease in extracellular sites (Périard et al., 2016).

Also, acute PE mobilizes high concentrations of CD8^+^ T cells (part of them from the skin) in response to the increase in catecholamines during its practice (Turner et al., 2016). This IS cells mobilization into the bloodstream during PE plays a significant role in removing hyper-reactive IS cells (Krüger and Mooren, 2014). For example, a session of high-intensity resistance exercise (60% 1RM; each set to concentric failure) is enough to induce temporary immunosuppression (i.e., a reduction in the ratio of CD4:CD8 T cells below 1:1; when the normal value is 1:4) (Jin et al., 2015). Also, 6-weeks (3x/week; 8–10 sets of 60s at 100% VO_2_peak) of high-intensity interval training (HIIT) can promote an anti-inflammatory state, attenuating the proliferation of a subset CD8^+^ T cell (CD8^low^, which produces high levels of IFN- γ and TNF-α) (Shiu, 2016). In addition, eight weeks of HIIT (5km running; work: rest ratio 1:1 ratio, 1 min. at 100% vVO_2_max interspersed with 1 min. of passive recovery) plus resistance training (4 sets of squats at 80% 1RM to concentric failure) promoted an increase in IL-10 and IL-6 (Monteiro et al., 2017). These studies suggest that if this training routine (high volume or high intensity) is sustained for weeks, it will induce a sustained IS alteration, triggering a state of temporary immunosuppression in response to this type of PE training (Shaw et al., 2017). Theoretically, this immunosuppression state imposed by high-load chronic PE can be seen as a protective factor to reduce the risk of developing autoimmune diseases (Shiu, 2016).

An interesting study observed that lifelong aerobic-trained individuals (with 40 ml⋅kg^–1^⋅min^–1^ VO_2_max) had higher blood Tregs markers concentrations (IL-10, forkhead box P3, and transforming growth factor-β), low ↓TNF-α/IL-10↑ ratio associated with lower memory CD8^+^ T cells when compared to sedentary counterparts (with 29 ml⋅kg^–1^⋅min^–1^ VO_2_max) (Minuzzi et al., 2017, 2018). Furthermore, IL-2, which is responsible for T cell proliferation and differentiation, and maturation of CD8^+^ memory T cells is decreased after chronic PE (Rhind et al., 1996). Notably, vitiligo patients have high IL-2 concentrations due to a high stimulation of T cells (Jian et al., 2014); and lower Tregs markers (IL-10 and forkhead box P3) (Lotti et al., 2015; Bhardwaj et al., 2020). A theoretical schematic model of the potential role of chronic PE on IS from vitiligo patients is pointed out in Figures 1B, 5.

### Potential Role of Chronic Physical Exercise on Mitochondrial Function and Structure: Implications on Vitiligo Development

**FIGURE 6:**
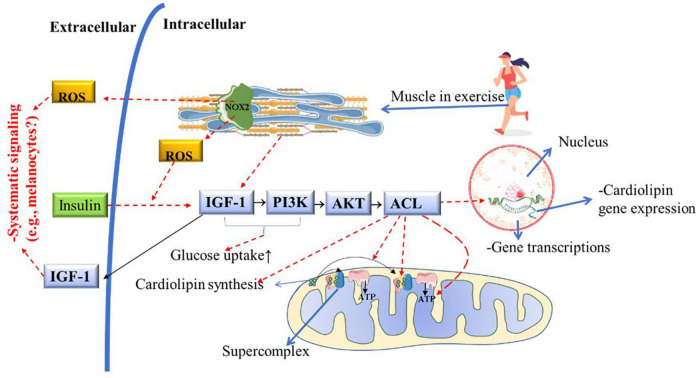
Potential role of chronic PE on cardiolipin metabolism, mitochondrial function, and structure in vitiligo patients. Muscle contraction during acute physical exercise activates and releases large amounts of IGF-1 into the bloodstream. Muscle contraction or insulin action is required to activate IGF-1 and the PI3K/AKT/ACL downstream metabolic pathway. ACL activation is necessary for cardiolipin synthesis and gene transcription. As a consequence of ACL activity, mitochondria increase the mitochondrial complexes and supercomplexes content and activity, enhancing ATP production (i.e., improving the Mitochondrial structure and function). Nox2 activation by acute physical exercise improves insulin-sensitive, increasing GLUT-4 translocation to the cellular membrane). This hypothetically facilitates the IGF-1 metabolic pathway downstream, consequently improving mitochondrial structure and function and preventing insulin resistance. ACL, Adenosine triphosphate-citrate lyase; ADP, adenosine diphosphate; AKT, protein kinase; GLUT4, glucose transport 4; IGF1, insulin-like growth factor-1; NOX2, NADPH oxidase isoform 2; PE, physical exercise; PI3K, phosphatidylinositol 3-kinase; ROS, reactive oxygen species.

Melanocytes and PBMCs from vitiligo patients have deficient energy metabolism due to the low concentration and abnormal distribution of cardiolipin in the mETC, which prevents the stability and creation of supercomplexes (Dell’Anna et al., 2007). Due to decreased cardiolipin metabolism, melanocytes from vitiligo patients have low energy production from glycolytic phosphorylation (due to defects in complex 1), resulting in low ATP production and high mitochondrial ROS production (Figure 2; Dell’Anna et al., 2017). Recently it was demonstrated that skeletal muscle increases mitochondrial cardiolipin quality and concentrations in response to IGF-1/PI3K/AKT/ACL pathway activation, which is induced by chronic physical aerobic exercise (Figure 6; Das et al., 2015). Interestingly, HIIT is a potent inducer of complex 1 enhancement in skeletal muscle (Bishop et al., 2014), leading to a greater mitochondrial ATP production and increase carbohydrate phosphorylation (Nilsson et al., 2019), which is impaired in melanocytes from vitiligo patients (Dell’Anna et al., 2017). On the other hand, in skeletal muscle, continuous aerobic-exercise training increases mitochondrial tissue volume (Bishop et al., 2014), which is a morphological change important to improve ROS clearance (Sansbury et al., 2011; Gifford et al., 2016).

**FIGURE 7:**
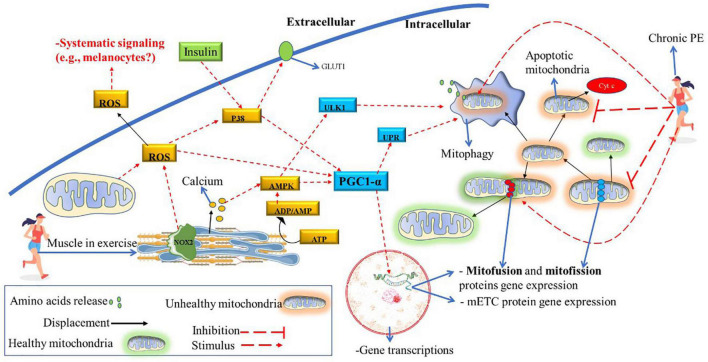
Potential role of chronic PE on mitochondrial function and structure. ATP degradation and calcium release caused by muscle contraction also significantly induce the PGC1-α and ULK1 downstream pathways. Also, muscle contraction stimulates large ROS released (by NOX2 and mitochondria activity), further activating the PGC1-α downstream. Acute PGC1-α activation results in gene transcription related to mitochondrial remodeling (mitofusin, mitofission, and mETC proteins) and mitophagy induction. Chronic PE will decrease the mitofission pathway and significantly enhance the mitofusion and mitophagy pathways. Consequently, the mitochondrial apoptosis pathway, which can induce cell death (due to the release of cytochrome C), decreases due to chronic PE. Acute PE also activates P38, an important stimulator of PGC1-α. For example, the lack of activity of this protein can decrease PGC1-α activation, even when there are other stimuli (e.g., only AMPK stimulation). Also, P38 is activated by insulin; in turn, p38 increases cell glucose uptake upregulating GLUT1. Hypothetically, a sedentary lifestyle and insulin resistance decrease the pulsative activation of p38, thus decreasing mitochondrial remodeling potential. ADP, adenosine diphosphate; AMP, adenosine monophosphate; AMPK, adenosine monophosphate-activated protein kinase; ATP, adenosine triphosphate; GLUT1, glucose transport 1; mETC, mitochondrial electron transport chain; NOX2, NADPH oxidase isoform 2; p38, mitogen-activated protein kinase; PE, physical exercise; PGC1-α, peroxisome proliferator-activated receptor γ coactivator-1α; ROS, reactive oxygen species; ULK1, Unc-51 Like Autophagy Activating Kinase 1; UPR, unfolded protein response.

In animal models developing T2D, a drastic decrease in cardiolipin levels in striated muscle was identified (Han et al., 2007). Therefore, hypothetically, insulin resistance (or T2D) may increase the risk of vitiligo developing in genetically predisposed individuals. In Figure 6, it is shown that PE improves insulin sensitivity via NOX2 (Carlos Henríquez-Olguín et al., 2019), and consequently, insulin signaling increases IGF-1activation (Das et al., 2015). It is well-known that chronic PE (HIIT) can reverse insulin resistance and the development of T2D and improve muscle mitochondrial capacity (Little et al., 2011). Therefore, future studies should investigate the relationship between insulin resistance and vitiligo development (if it is a cause-effect relationship or a common pathway). Also, the potential benefits of PE in improving insulin sensitivity and the IGF-1/PI3K/AKT/ACL metabolic pathway in vitiligo patients deserve further investigation. Moreover, both insulin and PE also activates P38 in skeletal muscle, an vital molecule to activate PGC1-α downstream pathway (see Figure 7) to promote mitochondrial remodeling resulting in improvement in glucose and fat metabolism (Hood et al., 2015). The pulsative activation of p38 in skeletal muscle is required for whole-body energy expenditures, which can prevent obesity and D2T (Bengal et al., 2020). However, P38 has a pleiotropic effect and opposite effect in different cell lines and cell environment (Stramucci et al., 2018). For instance, chronic high H_2_O_2_ levels can activate p38 continuously, inducing melanocytes to premature senescence (dysfunctional) and susceptible to apoptosis (Hou et al., 2022). The same pattern is found in skeletal muscle, the continuous p38 activation induces proinflammatory cytokines expression, insulin resistance (GLUT4 downregulation) and pathological muscle atrophy (Bengal et al., 2020). Therefore, further studies is needed to determine the role of chorionic PE on P38 activation and signaling in melanocyte of vitiligo development.

Vitiligo patients have a reduced mitophagy process in epidermal melanocytes (Ding et al., 2015). It is speculated that vitiligo is a systemic pathology (Lotti and D’Erme, 2014); however, it is not known whether this condition (decreased mitophagy) extends to other tissues. In the animal model (during acute PE), the impaired mitophagy process is characterized by low resistance to endurance exercise and a high metabolic acidosis (Vainshtein et al., 2015). Therefore, we can speculate that during acute PE, vitiligo patients produce higher lactate concentrations when compared to their healthy peers (with the same CRF values). It is also well established that PE (mainly aerobic type) is a potent inductor of the mitophagy process and mitochondrial turnover/remodeling (Hood et al., 2015; Tarpey et al., 2017). As chronic PE induces tissue changes that go beyond skeletal muscle tissue (e.g., increase in mitochondria in adipose tissue), it is plausible that changes such as improvement in mitochondrial metabolism (improves their structure and function) in the epidermis also could occur in response to physical training practice (see Figure 6). (Also, Table 1 summarizes the potential role of acute and chronic PE on vitiligo disease, which deserves future research in the epidermis of vitiligo patients).

## Conclusion and Future Directions

To the best of our knowledge, there are no studies in the literature that analyze the clinical applicability of chronic PE as a treatment strategy to improve vitiligo adverse events. According to the evidence discussed in this paper, we can hypothesize that chronic PE can modulate the etiological factors related to the onset of this condition which involves a high and chronic ROS production, a deficient EAS enzymatic activity, and a pro-inflammatory autoimmune response (Xie et al., 2016). Further, if confirmed, the improvement in these molecular mechanisms can lead to important clinical implications in vitiligo phenotypic traits and disease prognostic, that ultimately, may improve patient’s self-image, body confidence, and reduce psychological distress associated with this disease (Bonotis et al., 2016). Therefore, structured PE training may have a significant clinical impact not only in disease prognostic but also in patients’ quality of life.

The literature has shown that chronic PE plays a significant role in EAS enzymatic metabolism, IS modulation, and mitochondrial quality and function. However, more research is highly required from both observational and experimental studies to investigate the role of chronic PE training in vitiligo disease on four spheres: (i) disease prevention; (ii) during active disease development; (iii) during its stable/management phase; and (iv) during the repigmentation phase.

Observational studies comparing the general population with this clinical population should verify the relationship of daily physical activity with vitiligo development or severity. For instance, studies that investigate if CRF level or daily step count is associated with vitiligo’s disease. Further, experimental studies, in turn, should evaluate in more controlled settings, several aspects: (i) the role of acute and chronic PE in the *molecular mechanisms that are underlying the onset* of the disease and thus, test whether these positive changes in mitochondria, EAS enzymes and IS modulation reflects on the epidermis and in the melanocytes from vitiligo patients; (ii) test the *dose-response of different PE* types, volumes, and frequencies in vitiligo patients. Therefore, we suggest some clinically relevant markers that can be analyzed to test the potential applicability of chronic PE in vitiligo disease:

✓ Investigate if exercise training **improves EAS and decreases LP rates** in vitiligo patients.
✓ Investigate whether exercise training **improves the inflammatory profile** on vitiligo patients (i.e., the Th1/Th2 ratio), as well as extracellular Hsp70 concentrations.
✓ Check whether exercise training **alters the pigmentation/depigmentation process progression** in patients with vitiligo.
✓ Check whether the **energy metabolism** (respiratory quotient and plasma lactate concentrations) of patients with vitiligo differs from healthy peers, i.e., check whether these changes extend to muscle tissue. Currently, there are no studies associating the level of physical activity or CRF and the incidence of vitiligo in genetically susceptible individuals.
✓ Check whether PE training **alters the melanocytes mitochondria morphology** of vitiligo patients.

## Data Availability Statement

The original contributions presented in the study are included in the article/Supplementary Material, further inquiries can be directed to the corresponding author/s.

## Author Contributions

EF and EC designed the study and wrote the first draft. LB, RS, MDS, VH, AF, and RM added important intellectual content writing, criticizing, and correcting previous versions of the manuscript. All authors approved the final version of the manuscript.

## Conflict of Interest

The authors declare that the research was conducted in the absence of any commercial or financial relationships that could be construed as a potential conflict of interest.

## Publisher’s Note

All claims expressed in this article are solely those of the authors and do not necessarily represent those of their affiliated organizations, or those of the publisher, the editors and the reviewers. Any product that may be evaluated in this article, or claim that may be made by its manufacturer, is not guaranteed or endorsed by the publisher.

## References

[B1] Abbas A. K. Lichtman A. H. Pillai S. (2019a). *Immunologic Tolerance and Autoimmunity in Cellular and Molecular Immunology*, 9 Edn. Philadelphia: Elsevier.

[B2] Abbas A. K. Lichtman A. H. Pillai S. (2019b). *Innate Immunity Cellular and Molecular Immunology, Ninth Edition.* Philadelphia: Elsevier.

[B3] Adams V. Linke A. Kränkel N. Erbs S. Gielen S. Möbius-Winkler S. (2005). Impact of regular physical activity on the NAD (P) H oxidase and angiotensin receptor system in patients with coronary artery disease. *Circulation* 111 555–562. 10.1161/01.CIR.0000154560.88933.7E 15699275

[B4] Agarwala S. Malkud S. (2020). A study on the clinico-epidemiological profile of vitiligo patients and its association with endocrine, audiological and ocular abnormalities. *Iranian J. Dermatol.* 23 155–162. 10.22034/ijd.2020.120835

[B5] Aguiar S. S. Sousa C. V. Santos P. A. Barbosa L. P. Maciel L. A. Coelho-Júnior H. J. (2021). Master athletes have longer telomeres than age-matched non-athletes. A systematic review, meta-analysis and discussion of possible mechanisms. *Exp. Gerontol.* 146:111212. 10.1016/j.exger.2020.111212 33387607

[B6] Alizaei Yousefabadi H. Niyazi A. Alaee S. Fathi M. Mohammad Rahimi G. R. (2020). Anti-inflammatory effects of exercise on metabolic syndrome patients: a systematic review and meta-analysis. *Biol. Res. Nurs.* 23 280–292. 10.1177/1099800420958068 32938197

[B7] Alkhateeb A. Fain P. R. Thody A. Bennett D. C. Spritz R. A. (2003). Epidemiology of vitiligo and associated autoimmune diseases in caucasian probands and their families. *Pigment Cell Res.* 16 208–214. 10.1034/j.1600-0749.2003.00032.x 12753387

[B8] Antrobus M. Cuttell S. Machado L. R. (2021). “Chapter 8 - Epigenetics, exercise, and the immune system,” in *Epigenetics of Exercise and Sports*, ed. Raleigh S. M. (Cambridge, MA: Academic Press), 183–199.

[B9] Asea A. (2007). Mechanisms of HSP72 release. *J. Biosci.* 32 579–584. 10.1007/s12038-007-0057-5 17536177

[B10] Ataş H. Gönül M. (2017). Increased risk of metabolic syndrome in patients with vitiligo. *Balkan Med. J.* 34 219–225. 10.4274/balkanmedj.2016.1005 28443562 PMC5450861

[B11] Azzazi Y. Mostafa W. Z. Sayed K. S. Alhelf M. Safwat M. Mahrous A. (2021). Support for increased cardiovascular risk in non-segmental vitiligo among Egyptians: a hospital-based, case–control study. *Pigment Cell Melanoma Res.* 34 598–604. 10.1111/pcmr.12941 33098225

[B12] Barygina V. Becatti M. Lotti T. Moretti S. Taddei N. Fiorillo C. (2015). Treatment with low-dose cytokines reduces oxidative-mediated injury in perilesional keratinocytes from vitiligo skin. *J. Dermatol. Sci.* 79 163–170. 10.1016/j.jdermsci.2015.05.003 26051876

[B13] Bengal E. Aviram S. Hayek T. (2020). p38 MAPK in glucose metabolism of skeletal muscle: beneficial or harmful? *Int. J. Mol. Sci.* 21:6480. 10.3390/ijms21186480 32899870 PMC7555282

[B14] Bergqvist C. Ezzedine K. (2020). Vitiligo: a review. *Dermatology* 236 571–592. 10.1159/000506103 32155629

[B15] Bertolotti A. Boniface K. Vergier B. Mossalayi D. Taieb A. Ezzedine K. (2014). Type I Interferon signature in the initiation of the immune response in vitiligo. *Pigment Cell Melanoma Res.* 27 398–407. 10.1111/pcmr.12219 24438589

[B16] Bhardwaj S. Rani S. Kumaran M. S. Bhatia A. Parsad D. (2020). Expression of Th17- and Treg-specific transcription factors in vitiligo patients. *Int. J. Dermatol.* 59 474–481. 10.1111/ijd.14766 31909498

[B17] Bishop D. J. Granata C. Eynon N. (2014). Can we optimise the exercise training prescription to maximise improvements in mitochondria function and content? *Biochim. Biophys. Acta* 1840 1266–1275. 10.1016/j.bbagen.2013.10.012 24128929

[B18] Bonomini F. Rodella L. F. Rezzani R. (2015). Metabolic syndrome, aging and involvement of oxidative stress. *Aging Dis.* 6 109–120. 10.14336/AD.2014.0305 25821639 PMC4365955

[B19] Bonotis K. Pantelis K. Karaoulanis S. Katsimaglis C. Papaliaga M. Zafiriou E. (2016). Investigation of factors associated with health-related quality of life and psychological distress in vitiligo. *J. Dtsch. Dermatol. Ges.* 14 45–49. 10.1111/ddg.12729 26713637

[B20] Carlos Henríquez-Olguín S. B. Cabello-Verrugio C Jaimovich E Hidalgo E Jensen T. E. (2019). The emerging roles of nicotinamide adenine dinucleotide phosphate oxidase 2 in skeletal muscle redox signaling and metabolism. *Antioxid. Redox Signal.* 31 1371–1410. 10.1089/ars.2018.7678 31588777 PMC6859696

[B21] D’Arino A. Picardo M. Truglio M. Pacifico A. Iacovelli P. (2021). Metabolic comorbidities in vitiligo: a brief review and report of new data from a single-center experience. *Int. J. Mol. Sci.* 22:8820. 10.3390/ijms22168820 34445526 PMC8396221

[B22] Das S. Morvan F. Jourde B. Meier V. Kahle P. Brebbia P. (2015). ATP citrate lyase improves mitochondrial function in skeletal muscle. *Cell Metab.* 21 868–876. 10.1016/j.cmet.2015.05.006 26039450

[B23] de Sousa C. V. Sales M. M. Rosa T. S. Lewis J. E. de Andrade R. V. Simoes H. G. (2016). The antioxidant effect of exercise: a systematic review and meta-analysis. *Sports Med.* 47 277–293. 10.1007/s40279-016-0566-1 27260682

[B24] Dell’Anna M. L. Ottaviani M. Albanesi V. Vidolin A. P. Leone G. Ferraro C. (2007). Membrane lipid alterations as a possible basis for melanocyte degeneration in vitiligo. *J. Investig. Dermatol.* 127 1226–1233. 10.1038/sj.jid.5700700 17235326

[B25] Dell’Anna M. L. Ottaviani M. Bellei B. Albanesi V. Cossarizza A. Rossi L. (2010). Membrane lipid defects are responsible for the generation of reactive oxygen species in peripheral blood mononuclear cells from vitiligo patients. *J. Cell Physiol.* 223 187–193. 10.1002/jcp.22027 20049874

[B26] Dell’Anna M. L. Ottaviani M. Kovacs D. Mirabilii S. Brown D. A. Cota C. (2017). Energetic mitochondrial failing in vitiligo and possible rescue by cardiolipin. *Sci. Rep.* 7:13663. 10.1038/s41598-017-13961-5 29057950 PMC5654478

[B27] Dell’Anna M. L. Urbanelli S. Mastrofrancesco A. Camera E. Iacovelli P. Leone G. (2003). Alterations of mitochondria in peripheral blood mononuclear cells of vitiligo patients. *Pigment Cell Melanoma Res.* 16 553–559. 10.1034/j.1600-0749.2003.00087.x 12950736

[B28] Demirbaş A. Elmas ÖF. Atasoy M. Türsen Ü Lotti T. (2021). Can monocyte to HDL cholesterol ratio and monocyte to lymphocyte ratio be markers for inflammation and oxidative stress in patients with vitiligo? A preliminary study. *Arch. Dermatol. Res.* 313 491–498. 10.1007/s00403-020-02129-3 32816078

[B29] Deng R. Zhang P. Liu W. Zeng X. Ma X. Shi L. (2018). HDAC is indispensable for IFN-γ-induced B7-H1 expression in gastric cancer. *Clin. Epigenet.* 10:153. 10.1186/s13148-018-0589-6 30537988 PMC6288935

[B30] Dimauro I. Paronetto M. P. Caporossi D. (2020). Exercise, redox homeostasis and the epigenetic landscape. *Redox Biol.* 35:101477. 10.1016/j.redox.2020.101477 32127290 PMC7284912

[B31] Ding G.-Z. Zhao W.-E. Li X. Gong Q.-L. Lu Y. (2015). A comparative study of mitochondrial ultrastructure in melanocytes from perilesional vitiligo skin and perilesional halo nevi skin. *Arch. Dermatol. Res.* 307 281–289. 10.1007/s00403-015-1544-4 25672813

[B32] Dwivedi M. Laddha N. C. Shah K. Shah B. J. Begum R. (2013). Involvement of interferon-gamma genetic variants and intercellular adhesion molecule-1 in onset and progression of generalized vitiligo. *J. Interferon Cytokine Res.* 33 646–659. 10.1089/jir.2012.0171 23777204 PMC3814581

[B33] Edwardson C. L. Gorely T. Davies M. J. Gray L. J. Khunti K. Wilmot E. G. (2012). Association of sedentary behaviour with metabolic syndrome: a meta-analysis. *PLoS One* 7:e34916. 10.1371/journal.pone.0034916 22514690 PMC3325927

[B34] Ellingsgaard H. Hojman P. Pedersen B. K. (2019). Exercise and health — emerging roles of IL-6. *Curr. Opin. Physiol.* 10 49–54. 10.1016/j.cophys.2019.03.009

[B35] Farrell S. W. Finley C. E. Grundy S. M. (2012). Cardiorespiratory fitness, LDL cholesterol, and CHD mortality in men. *Med. Sci. Sports Exerc.* 44 2132–2137. 10.1249/MSS.0b013e31826524be 22776869

[B36] Gafter U. Malachi T. Ori Y. Breitbart H. (1997). The role of calcium in human lymphocyte DNA repair ability. *J. Lab. Clin. Med.* 130 33–41. 10.1016/S0022-2143(97)90056-1 9242364

[B37] Gifford J. R. Garten R. S. Nelson A. D. Trinity J. D. Layec G. Witman M. A. (2016). Symmorphosis and skeletal muscle V̇O2 max: in vivo and in vitro measures reveal differing constraints in the exercise-trained and untrained human. *J. Physiol.* 594 1741–1751. 10.1113/JP271229 26614395 PMC4799962

[B38] Giri P. S. Dwivedi M. Laddha N. C. Begum R. Bharti A. H. (2020). Altered expression of nuclear factor of activated T cells, forkhead box P3, and immune-suppressive genes in regulatory T cells of generalized vitiligo patients. *Pigment Cell Melanoma Res.* 33 566–578. 10.1111/pcmr.12862 31917889

[B39] Gjevestad G. O. Holven K. B. Ulven S. M. (2015). Effects of exercise on gene expression of inflammatory markers in human peripheral blood cells: a systematic review. *Curr. Cardiovasc. Risk Rep.* 9 1–17. 10.1007/s12170-015-0463-4 26005511 PMC4439514

[B40] Glassman S. J. (2014). “ROS and vitiligo,” in *Systems Biology of Free Radicals and Antioxidants*, ed. Laher I. (Berlin: Springer), 3677–3695.

[B41] Gounder S. S. Kannan S. Devadoss D. Miller C. J. Whitehead K. S. Odelberg S. J. (2012). Impaired transcriptional activity of Nrf2 in age-related myocardial oxidative stress is reversible by moderate exercise training. *PLoS One* 7:e45697. 10.1371/journal.pone.0045697 23029187 PMC3454427

[B42] Han X. Yang J. Yang K. Zhao Z. Abendschein D. R. Gross R. W. (2007). Alterations in myocardial cardiolipin content and composition occur at the very earliest stages of diabetes: a shotgun lipidomics study. *Biochemistry* 46 6417–6428. 10.1021/bi7004015 17487985 PMC2139909

[B43] Hasan R. Agarwal K. Podder I. Misitzis A. Schwartz R. A. Wollina U. (2021). Simvastatin in vitiligo: an update with recent review of the literature. *Int. J. Dermatol.* 60 e390–e396. 10.1111/ijd.15330 33554328

[B44] Hauff K. D. Choi S.-Y. Frohman M. A. Hatch G. M. (2009). Cardiolipin synthesis is required to support human cholesterol biosynthesis from palmitate upon serum removal in Hela cells. *Can. J. Physiol. Pharmacol.* 87 813–820. 10.1139/Y09-055 19898564 PMC4125688

[B45] Henríquez-Olguín C. Díaz-Vegas A. Utreras-Mendoza Y. Campos C. Arias-Calderón M. Llanos P. (2016). NOX2 inhibition impairs early muscle gene expression induced by a single exercise bout. *Front. Physiol.* 7:282. 10.3389/fphys.2016.00282 27471471 PMC4944119

[B46] Hood D. A. Tryon L. D. Vainshtein A. Memme J. Chen C. Pauly M. (2015). Exercise and the regulation of mitochondrial turnover. *Prog. Mol. Biol. Transl. Sci.* 135 99–127. 10.1016/bs.pmbts.2015.07.007 26477912

[B47] Horsburgh S. Todryk S. Toms C. Moran C. N. Ansley L. (2015). Exercise-conditioned plasma attenuates nuclear concentrations of DNA methyltransferase 3B in human peripheral blood mononuclear cells. *Physiol. Rep.* 3:e12621. 10.14814/phy2.12621 26660547 PMC4760429

[B48] Hou X. Shi J. Sun L. Song L. Zhao W. Xiong X. (2022). The involvement of ERK1/2 and p38 MAPK in the premature senescence of melanocytes induced by H2O2 through a p53-independent p21 pathway. *J. Dermatol. Sci.* Online ahead of print., 10.1016/j.jdermsci.2022.01.002 35042627

[B49] Jenkins R. Goldfarb A. (1993). Introduction: oxidant stress, aging, and exercise. *Med. Sci. Sports Exerc.* 25 210–212. 8450723

[B50] Jian Z. Li K. Song P. Zhu G. Zhu L. Cui T. (2014). Impaired activation of the Nrf2-are signaling pathway undermines H2O2-induced oxidative stress response: a possible mechanism for melanocyte degeneration in vitiligo. *J. Investig. Dermatol.* 134 2221–2230. 10.1038/jid.2014.152 24662764

[B51] Jin C.-H. Paik I.-Y. Kwak Y.-S. Jee Y.-S. Kim J.-Y. (2015). Exhaustive submaximal endurance and resistance exercises induce temporary immunosuppression via physical and oxidative stress. *J. Exerc. Rehabil.* 11:198. 10.12965/jer.150221 26331134 PMC4548676

[B52] Jin Y. Mailloux C. M. Gowan K. Riccardi S. L. LaBerge G. Bennett D. C. (2007). NALP1 in vitiligo-associated multiple autoimmune disease. *New England J. Med.* 356 1216–1225. 10.1056/NEJMoa061592 17377159

[B53] Karadag A. S. Tutal E. Ertugrul D. T. (2011). Insulin resistance is increased in patients with vitiligo. *Acta Dermato Venereologica* 91 541–544. 10.2340/00015555-1141 21597678

[B54] Kocer B. Nazliel B. Oztas M. Batur H. (2009). Vitiligo and multiple sclerosis in a patient treated with interferon beta-1a: a case report. *Eur. J. Neurol.* 16 e78–e79. 10.1111/j.1468-1331.2009.02563.x 19222549

[B55] Krüger C. Schallreuter K. U. (2012). A review of the worldwide prevalence of vitiligo in children/adolescents and adults. *Int. J. Dermatol.* 51 1206–1212. 10.1111/j.1365-4632.2011.05377.x 22458952

[B56] Krüger C. Schallreuter K. U. (2013). Cumulative life course impairment in vitiligo. *Curr. Probl. Dermatol.* 44 102–117. 10.1159/000350010 23796814

[B57] Krüger K. Mooren F. C. (2014). Exercise-induced leukocyte apoptosis. *Exerc. Immunol. Rev.* 20 117–134. 24974724

[B58] Laddha N. C. Dwivedi M. Gani A. R. Shajil E. Begum R. (2013). Involvement of superoxide dismutase isoenzymes and their genetic variants in progression of and higher susceptibility to vitiligo. *Free Radic. Biol. Med.* 65 1110–1125. 10.1016/j.freeradbiomed.2013.08.189 24036105

[B59] Laddha N. C. Dwivedi M. Mansuri M. S. Singh M. Gani A. R. Yeola A. P. (2014). Role of oxidative stress and autoimmunity in onset and progression of vitiligo. *Exp. Dermatol.* 23 352–353. 10.1111/exd.12372 24628992

[B60] Le Poole I. C. Mehrotra S. (2017). Replenishing regulatory T cells to halt depigmentation in vitiligo. *J. Investig. Dermatol. Symp. Proc.* 18 S38–S45. 10.1016/j.jisp.2016.10.023 28941492

[B61] Lee A.-Y. Youm Y.-H. Kim N.-H. Yang H. Choi W.-I. (2004). Keratinocytes in the depigmented epidermis of vitiligo are more vulnerable to trauma (suction) than keratinocytes in the normally pigmented epidermis, resulting in their apoptosis. *Br. J. Dermatol.* 151 995–1003. 10.1111/j.1365-2133.2004.06136.x 15541077

[B62] Lee E. Muñoz C. McDermott B. Beasley K. Yamamoto L. Hom L. (2015). Extracellular and cellular Hsp72 differ as biomarkers in acute exercise/environmental stress and recovery. *Scand. J. Med. Sci. Sports* 27 66–74. 10.1111/sms.12621 26643874

[B63] Leeuwenburgh C. Heinecke J. (2001). Oxidative stress and antioxidants in exercise. *Curr. Med. Chem.* 8 829–838. 10.2174/0929867013372896 11375753

[B64] Lionaki E. Markaki M. Palikaras K. Tavernarakis N. (2015). Mitochondria, autophagy and age-associated neurodegenerative diseases: new insights into a complex interplay. *Biochim. Biophys. Acta* 1847 1412–1423. 10.1016/j.bbabio.2015.04.010 25917894

[B65] Lira F. S. Rosa J. C. Lima-Silva A. E. Souza H. A. Caperuto E. C. Seelaender M. C. (2010). Sedentary subjects have higher PAI-1 and lipoproteins levels than highly trained athletes. *Diabetol. Metab. Syndr.* 2:7. 10.1186/1758-5996-2-7 20205861 PMC2826310

[B66] Little J. P. Gillen J. B. Percival M. E. Safdar A. Tarnopolsky M. A. Punthakee Z. (2011). Low-volume high-intensity interval training reduces hyperglycemia and increases muscle mitochondrial capacity in patients with type 2 diabetes. *J. Appl. Physiol.* 111 1554–1560. 10.1152/japplphysiol.00921.2011 21868679

[B67] Lotti T. D’Erme A. M. (2014). Vitiligo as a systemic disease. *Clin. Dermatol.* 32 430–434. 24767192 10.1016/j.clindermatol.2013.11.011

[B68] Lotti T. Hercogova J. Fabrizi G. (2015). Advances in the treatment options for vitiligo: activated low-dose cytokines-based therapy. *Exp. Opin. Pharmacother.* 16 2485–2496. 10.1517/14656566.2015.1087508 26372794

[B69] Lu J. Holmgren A. (2014). The thioredoxin antioxidant system. *Free Radic. Biol. Med.* 66 75–87. 23899494 10.1016/j.freeradbiomed.2013.07.036

[B70] Lu Z. Xu Y. Song Y. Bíró I. Gu Y. (2021). A mixed comparisons of different intensities and types of physical exercise in patients with diseases related to oxidative stress: a systematic review and network meta-analysis. *Front. Physiol.* 12:700055. 10.3389/fphys.2021.700055 34421637 PMC8375596

[B71] Ma S.-H. Ang M.-D. Chang Y.-T. Dai Y.-X. (2021). Association between vitiligo and hearing loss: a systemic review and metaanalysis. *J. Am. Acad. Dermatol.* 85 1465–1472. 10.1016/j.jaad.2020.12.029 33359081

[B72] Maresca V. Roccella M. Roccella F. Camera E. Del Porto G. Passi S. (1997). Increased sensitivity to peroxidative agents as a possible pathogenic factor of melanocyte damage in vitiligo. *J. Investig. Dermatol.* 109 310–313. 10.1111/1523-1747.ep12335801 9284096

[B73] Margaritelis N. V. Theodorou A. A. Paschalis V. Veskoukis A. S. Dipla K. Zafeiridis A. (2018). Adaptations to endurance training depend on exercise-induced oxidative stress: exploiting redox interindividual variability. *Acta Physiol.* 222:12898. 10.1111/apha.12898 28544643

[B74] Martins C. Migayron L. Drullion C. Jacquemin C. Lucchese F. Rambert J. (2021). Vitiligo skin T cells are prone to produce type 1 and type 2 cytokines to induce melanocyte dysfunction and epidermal inflammatory response through jak signaling. *J. Invest. Dermatol*. Online ahead of print., 10.1016/j.jid.2021.09.015, 34655610

[B75] Minuzzi L. G. Rama L. Bishop N. C. Rosado F. Martinho A. Paiva A. (2017). Lifelong training improves anti-inflammatory environment and maintains the number of regulatory T cells in masters athletes. *Eur. J. Appl. Physiol.* 117 1131–1140. 10.1007/s00421-017-3600-6 28391394

[B76] Minuzzi L. G. Rama L. Chupel M. U. Rosado F. Dos Santos J. V. Simpson R. (2018). Effects of lifelong training on senescence and mobilization of T lymphocytes in response to acute exercise. *Exerc. Immunol. Rev.* 24 72–84. 29461967

[B77] Monteiro P. A. Campos E. Z. de Oliveira F. P. Peres F. P. Rosa-Neto J. C. Pimentel G. D. (2017). Modulation of inflammatory response arising from high-intensity intermittent and concurrent strength training in physically active males. *Cytokine* 91 104–109. 10.1016/j.cyto.2016.12.007 28043028

[B78] Mosenson J. A. (2013). *Defining a Role for Inducible Heat Shock Protein 70 (Hsp70i) in Mediating Autoimmune Vitiligo*. Ph.D. thesis, Doctoral dissertation, Loyola University Chicago, Chicago.

[B79] Muthusamy V. R. Kannan S. Sadhaasivam K. Gounder S. S. Davidson C. J. Boeheme C. (2012). Acute exercise stress activates Nrf2/ARE signaling and promotes antioxidant mechanisms in the myocardium. *Free Radic. Biol. Med.* 52 366–376. 10.1016/j.freeradbiomed.2011.10.440 22051043 PMC3800165

[B80] Namazi N. Amani M. Haghighatkhah H. R. Noori E. Abdollahimajd F. (2021). Increased risk of subclinical atherosclerosis and metabolic syndrome in patients with vitiligo: a real association or a coincidence? *Dermatol. Ther.* 34:e14803. 10.1111/dth.14803 33496053

[B81] Natarajan V. T. Ganju P. Singh A. Vijayan V. Kirty K. Yadav S. (2014). IFN-γ signaling maintains skin pigmentation homeostasis through regulation of melanosome maturation. *Proc. Natl. Acad. Sci.* 111 2301–2306. 10.1073/pnas.1304988111 24474804 PMC3926048

[B82] Ni J. Qiu L.-J. Zhang M. Wen P.-F. Ye X.-R. Liang Y. (2014). CTLA-4 CT60 (rs3087243) polymorphism and autoimmune thyroid diseases susceptibility: a comprehensive meta-analysis. *Endocr. Res.* 39 180–188. 10.3109/07435800.2013.879167 24697361

[B83] Nilsson A. Björnson E. Flockhart M. Larsen F. J. Nielsen J. (2019). Complex I is bypassed during high intensity exercise. *Nat. Commun.* 10 1–11. 10.1038/s41467-019-12934-8 31699973 PMC6838197

[B84] Ortona E. Maselli A. Delunardo F. Colasanti T. Giovannetti A. Pierdominici M. (2014). Relationship between redox status and cell fate in immunity and autoimmunity. *Antioxid. Redox Signal.* 21 103–122. 10.1089/ars.2013.5752 24359147

[B85] Pannala V. R. Dash R. K. (2014). Mechanistic characterization of the thioredoxin system in the removal of hydrogen peroxide. *Free Radic. Biol. Med.* 78 42–55. 10.1016/j.freeradbiomed.2014.10.508 25451645 PMC4280359

[B86] Pareja-Galeano H. Sanchis-Gomar F. García-Giménez J. L. (2014). Physical exercise and epigenetic modulation: elucidating intricate mechanisms. *Sports Med.* 44 429–436. 10.1007/s40279-013-0138-6 24399634

[B87] Périard J. D. Travers G. J. S. Racinais S. Sawka M. N. (2016). Cardiovascular adaptations supporting human exercise-heat acclimation. *Auton. Neurosci.* 196 52–62. 10.1016/j.autneu.2016.02.002 26905458

[B88] Qi J. Luo X. Ma Z. Zhang B. Li S. Duan X. (2020). Swimming exercise protects against insulin resistance via regulating oxidative stress through nox4 and akt signaling in high-fat diet-fed mice. *J. Diabetes Res.* 2020:2521590. 10.1155/2020/2521590 32051831 PMC6995488

[B89] Radak Z. Hart N. Marton O. Koltai E. (2014). “Regular exercise results in systemic adaptation against oxidative stress,” in *Systems Biology of Free Radicals and Antioxidants*, ed. Laher I. (Berlin: Springer), 3855–3869. 10.1055/s-0029-1233464

[B90] Rashighi M. Agarwal P. Richmond J. M. Harris T. H. Dresser K. Su M.-W. (2014). CXCL10 is critical for the progression and maintenance of depigmentation in a mouse model of vitiligo. *Sci. Transl. Med.* 6:223ra223. 10.1126/scitranslmed.3007811 PMC4086941 24523323

[B91] Rhind S. G. Shek P. N. Shinkai S. Shephard R. J. (1996). Effects of moderate endurance exercise and training on in vitro lymphocyte proliferation, interleukin-2 (IL-2) production, and IL-2 receptor expression. *Eur. J. Appl. Physiol. Occup. Physiol.* 74 348–360. 10.1007/bf02226932 8911828

[B92] Riding R. L. Harris J. E. (2019). The role of memory CD8+ T cells in vitiligo. *J. Immunol.* 203 11–19. 10.4049/jimmunol.1900027 31209143 PMC6709670

[B93] Sansbury B. E. Jones S. P. Riggs D. W. Darley-Usmar V. M. Hill B. G. (2011). Bioenergetic function in cardiovascular cells: the importance of the reserve capacity and its biological regulation. *Chem. Biol. Interactions* 191 288–295. 10.1016/j.cbi.2010.12.002 21147079 PMC3090710

[B94] Schallreuter K. Pittelkow M. Wood J. (1991a). Defects in antioxidant defense and calcium transport in the epidermis of xeroderma pigmentosum patients. *Arch. Dermatol. Res.* 283 449–455. 10.1007/BF00371781 1801654

[B95] Schallreuter K. U. Wood J. M. Berger J. (1991b). Low catalase levels in the epidermis of patients with vitiligo. *J. Investig. Dermatol.* 97 1081–1085. 10.1111/1523-1747.ep12492612 1748819

[B96] Schallreuter K. U. (2014). “Reactive oxygen species and reactive nitrogen species in vitiligo,” in *Systems Biology of Free Radicals and Antioxidants*, ed. Laher I. (Berlin: Springer), 3697–3736.

[B97] Schallreuter K. U. Salem M. A. E. L. Gibbons N. C. J. Martinez A. Slominski R. Lüdemann J. (2012). Blunted epidermal l-tryptophan metabolism in vitiligo affects immune response and ROS scavenging by Fenton chemistry, part 1: epidermal H2O2/ONOO–mediated stress abrogates tryptophan hydroxylase and dopa decarboxylase activities, leading to low serotonin and melatonin levels. *FASEB J.* 26 2457–2470. 10.1096/fj.11-197137 22415302

[B98] Schallreuter K. U. Wood J. M. (1991). Sensitivity and resistance in human metastatic melanoma to the new chloroethylnitrosourea anti-tumor drug fotemustine. *Biochim. Biophys. Acta* 1096 277–283. 10.1016/0925-4439(91)90063-f 2065101

[B99] Schallreuter-Wood K. U. Pittelkow M. R. Swanson N. N. (1996). Defective calcium transport in vitiliginous melanocytes. *Arch. Dermatol. Res.* 288 11–13. 10.1007/BF02505036 8750928

[B100] Schlame M. Greenberg M. L. (2017). Biosynthesis, remodeling and turnover of mitochondrial cardiolipin. *Biochim. Biophys. Acta* 1862 3–7. 10.1016/j.bbalip.2016.08.010 27556952 PMC5125896

[B101] Shajil E. Begum R. (2006). Antioxidant status of segmental and non-segmental vitiligo. *Pigment Cell Res.* 19 179–180. 10.1111/j.1600-0749.2006.00299.x 16524434

[B102] Sharma L. K. Lu J. Bai Y. (2009). Mitochondrial respiratory complex I: structure, function and implication in human diseases. *Curr. Med. Chem.* 16 1266–1277. 10.2174/092986709787846578 19355884 PMC4706149

[B103] Sharma Y. K. Bansal P. Menon S. Prakash N. (2017). Metabolic syndrome in vitiligo patients among a semi-urban Maharashtrian population: a case control study. *Diabetes Metab. Syndr.* 11 S77–S80. 10.1016/j.dsx.2016.12.009 28017282

[B104] Shaw D. M. Merien F. Braakhuis A. Dulson D. (2017). T-cells and their cytokine production: the anti-inflammatory and immunosuppressive effects of strenuous exercise. *Cytokine* 104 136–142. 10.1016/j.cyto.2017.10.001 29021092

[B105] Shi Q. Zhang W. Guo S. Jian Z. Li S. Li K. (2016). Oxidative stress-induced overexpression of miR-25: the mechanism underlying the degeneration of melanocytes in vitiligo. *Cell Death Differentiation* 23 496–508. 10.1038/cdd.2015.117 26315342 PMC5072443

[B106] Shi Y.-L. Weiland M. Li J. Hamzavi I. Henderson M. Huggins R. H. (2013). MicroRNA expression profiling identifies potential serum biomarkers for non-segmental vitiligo. *Pigment Cell Melanoma Res.* 26 418–421. 10.1111/pcmr.12086 23470042

[B107] Shiu M. (2016). *Modulation of T Cell Distribution and Function by High-Intensity Interval Training.* Toronto, ON: University of Toronto.

[B108] Skurkovich B. Skurkovich S. (2006). “Inhibition of IFN-γ as a method of treatment of various autoimmune diseases, including skin diseases,” in *Cytokines as Potential Therapeutic Targets for Inflammatory Skin Diseases*, eds Numerof R. Dinarello C. A. Asadullah K. (Berlin: Springer), 1–27. 10.1007/3-540-37673-9_1

[B109] Song G. G. Kim J.-H. Lee Y. H. (2013). The CTLA-4 +49A/G, CT60A/G and PTPN22 1858 C/T polymorphisms and susceptibility to vitiligo: a meta-analysis. *Mol. Biol. Rep.* 40 2985–2993. 10.1007/s11033-012-2370-9 23264102

[B110] Stramucci L. Pranteda A. Bossi G. (2018). Insights of crosstalk between p53 protein and the mkk3/mkk6/p38 mapk signaling pathway in cancer. *Cancers* 10:131. 10.3390/cancers10050131 29751559 PMC5977104

[B111] Tanacan E. Atakan N. (2020). Higher incidence of metabolic syndrome components in vitiligo patients: a prospective cross-sectional study✩,✩✩. *An. Bras. Dermatol.* 95 165–172. 10.1016/j.abd.2019.07.006 32113676 PMC7175042

[B112] Tarpey M. D. Davy K. P. McMillan R. P. Bowser S. M. Halliday T. M. Boutagy N. E. (2017). Skeletal muscle autophagy and mitophagy in endurance-trained runners before and after a high-fat meal. *Mol. Metab.* 6 1597–1609. 10.1016/j.molmet.2017.10.006 29097020 PMC5699914

[B113] Teulings H. Overkamp M. Ceylan E. Nieuweboer-Krobotova L. Bos J. Nijsten T. (2013). Decreased risk of melanoma and nonmelanoma skin cancer in patients with vitiligo: a survey among 1307 patients and their partners. *Br. J. Dermatol.* 168 162–171. 10.1111/bjd.12111 23136900

[B114] Tsai M.-F. Jiang D. Zhao L. Clapham D. Miller C. (2014). Functional reconstitution of the mitochondrial Ca2+/H+ antiporter Letm1. *J. Gen. Physiol.* 143 67–73. 10.1085/jgp.201311096 24344246 PMC3874562

[B115] Turner J. E. Wadley A. J. Aldred S. Fisher J. P. Bosch J. A. Campbell J. P. (2016). Intensive exercise does not preferentially mobilize skin-homing T cells and NK cells. *Med. Sci. Sports Exerc.* 48 1285–1293. 10.1249/MSS.0000000000000914 26918560

[B116] Vainshtein A. Tryon L. D. Pauly M. Hood D. A. (2015). Role of PGC-1α during acute exercise-induced autophagy and mitophagy in skeletal muscle. *Am. J. Physiol. Cell Physiol.* 308 C710–C719. 10.1152/ajpcell.00380.2014 25673772 PMC4420796

[B117] Vargas-Mendoza N. Morales-González Á Madrigal-Santillán E. O. Madrigal-Bujaidar E. Álvarez-González I. García-Melo L. F. (2019). Antioxidant and adaptative response mediated by nrf2 during physical exercise. *Antioxidants* 8:196. 10.3390/antiox8060196 31242588 PMC6617290

[B118] Venditti P. Masullo P. Di Meo S. (1999). Effect of training on H2 O2 release by mitochondria from rat skeletal muscle. *Arch. Biochem. Biophys.* 372 315–320. 10600170 10.1006/abbi.1999.1494

[B119] Verma D. Hussain K. Namiq K. S. Firoz A. Bouchama M. Raza M. (2021). Vitiligo: the association with metabolic syndrome and the role of simvastatin as an immunomodulator. *Cureus* 13:e14029. 10.7759/cureus.14029 33898117 PMC8059484

[B120] Wang Y. Wang K. Liang J. Yang H. Dang N. Yang X. (2015). Differential expression analysis of miRNA in peripheral blood mononuclear cells of patients with non-segmental vitiligo. *J. Dermatol.* 42 193–197. 10.1111/1346-8138.12725 25495156

[B121] Xie H. Zhou F. Liu L. Zhu G. Li Q. Li C. (2016). Vitiligo: how do oxidative stress-induced autoantigens trigger autoimmunity? *J. Dermatol. Sci.* 81 3–9. 10.1016/j.jdermsci.2015.09.003 26387449

[B122] You S. Cho Y. H. Byun J. S. Shin E. C. (2013). Melanocyte-specific CD8+ T cells are associated with epidermal depigmentation in a novel mouse model of vitiligo. *Clin. Exp. Immunol.* 174 38–44. 10.1111/cei.12146 23711243 PMC3784211

[B123] Zhang Y. Liu L. Jin L. Yi X. Dang E. Yang Y. (2014). Oxidative stress–induced calreticulin expression and translocation: new insights into the destruction of melanocytes. *J. Investig. Dermatol.* 134 183–191. 10.1038/jid.2013.268 23771121

[B124] Zhang Z. Yang X. Liu O. Cao X. Tong J. Xie T. (2021). Differentially expressed microRNAs in peripheral blood mononuclear cells of non-segmental vitiligo and their clinical significance. *J. Clin. Lab. Anal.* 35:e23648. 10.1002/jcla.23648 33169883 PMC7891539

[B125] Zhao M. Gao F. Wu X. Tang J. Lu Q. (2010). Abnormal DNA methylation in peripheral blood mononuclear cells from patients with vitiligo. *Br. J. Dermatol.* 163 736–742. 10.1111/j.1365-2133.2010.09919.x 20560952

[B126] Zhou L. Lim H. W. Mi Q.-S. (2019). “Epigenetics,” in *Vitiligo*, eds Picardo M. Taïeb A. (Cham: Springer International Publishing), 253–264.

