# Supplementary material for: Potential Role of Chronic Physical Exercise as a Treatment in the Development of Vitiligo
Source: Front Physiol. 2022 Mar 10;13:843784. doi: 10.3389/fphys.2022.843784 (PMC8960951; doi:10.3389/fphys.2022.843784)
Supplement: Supplementary file 1 [file Table_1.docx]

**Table S1:** The potential role of physical exercise training on vitiligo patients.

| **Modifiable vitiligo profile** | **Potential from acute exercise** | **Potential from physical training** |
| --- | --- | --- |
| **Metabolic profile** | | |
| Associated to MetS[[1-4](#_ENREF_1)]and ↑ insulin resistance, adipose tissue, blood pressure, LDL-C, and ↓ HDL-C[[1](#_ENREF_1), [3-9](#_ENREF_3)]. | ↑ gene transcription to glucose uptake, fat oxidation, cardiac and vascular remodeling[[10-12](#_ENREF_10)]. | ↓ insulin resistance[[13](#_ENREF_13), [14](#_ENREF_14)].  ↓ LDL-C[[15](#_ENREF_15)] and blood pressure↑ HDL-C [[16](#_ENREF_16)].  Improvement in severalMetS markers[[16](#_ENREF_16), [17](#_ENREF_17)].  ↓ adipose tissue[[18](#_ENREF_18)]. |
| **Redox System profile** | | |
| ↑ ROS/RNS[[19](#_ENREF_19)].  ↓ EAS enzyme (GPx, TrxR, and CAT)[[20-22](#_ENREF_20)].  Chronic ↑NADPH-oxidase and SOD activity[[23](#_ENREF_23), [24](#_ENREF_24)].  ↑Lipid, protein and DNA peroxidation[[23](#_ENREF_23), [25](#_ENREF_25)]. | Acute ↑NADPH-oxidase activity induces EAS enzymes gene transcription(via NF-κB pathway)[[26](#_ENREF_26)].  ↑ Nrf2-ARE/HO-1 pathway activation [[27](#_ENREF_27)]. | ↑ EAS enzymes capacity and ↓ lipid, protein, and DNA peroxidation[[28](#_ENREF_28), [29](#_ENREF_29)].  ↓ROS[[30](#_ENREF_30)].  ↓ NADPH-oxidase activity induced ROS [[31](#_ENREF_31)].  ↑ NADPH synthesis [[32](#_ENREF_32)]. |
| **Mitochondrial structure and function profile** | | |
| ↓ mitochondrial mitophagy in melanocytes[[33](#_ENREF_33)].  ↓ cardiolipin quality andquantity; ↓melanocytes mitochondrialATP production andmitochondrial complexes and supercomplex activity.↑ mitochondrial ROS emission [[34-37](#_ENREF_34)]. | ↑ gene transcription for mitochondrial biogenesis and remodeling. [[26](#_ENREF_26), [38](#_ENREF_38), [39](#_ENREF_39)].  ↑ IGF-1/PI3K/AKT/ACL pathway activation to cardiolipin biosynthesis[[40](#_ENREF_40)]. | ↑ mitochondrial mitophagy[[41](#_ENREF_41)] and remodeling (mitofision,mitofusion)[[42](#_ENREF_42)].  ↑ ATP mitochondrial (increase in complex 1) and mitochondrial mass [[39](#_ENREF_39)].  ↑ cardiolipin content and supercomplex formation and ATP content[[40](#_ENREF_40)].  ↓ mitochondrial ROS emission [[43](#_ENREF_43)]. |
| **Immune function profile** | | |
| ↑ IL-2, IL-6, and IL-15;↓ IL-4 and IL-10;↑ TNF-α, IFN-α, and IFN- γ;↑ memory CD8^+^ T cell;↓Tregs[[44](#_ENREF_44), [45](#_ENREF_45)].  ↑ extracellular HSP[[46](#_ENREF_46)]. | CD8^+^ T cells mobilization to bloodstream removing hyper-reactive senescent cells[[47](#_ENREF_47)].  ↑ acute increase in, IFNα, IL-6, IL-1β inducing regulatory effect inIL-4, IL-10, and IL-1RA[[48](#_ENREF_48)]. | ↓ IL-2 [[49](#_ENREF_49)],IL-6[[50](#_ENREF_50)], and↑ IL-15 [[51](#_ENREF_51)].  ↑ IL-10[[52](#_ENREF_52)] andIL-4 [[51](#_ENREF_51), [53](#_ENREF_53)].  ↓ TNF-α[[52](#_ENREF_52)] and IFN- γ[[54](#_ENREF_54)].  ↓total lymphocytes [[55](#_ENREF_55), [56](#_ENREF_56)],CD8^+^ T cell proliferation [[57](#_ENREF_57)], memory CD8^+^ T cell[[58](#_ENREF_58)] and ↑ senescent CD8^+^ T cell apoptosis [[59](#_ENREF_59)].  ↑Tregs[[60-62](#_ENREF_60)].  ↓ extracellular HSP and ↑ intracellular HSP [[63](#_ENREF_63)] |

Acronyms: ↑, increase; ↓, decrease;ATP, adenosine triphosphate; CAT, catalase; EAS, endogenous antioxidant system;GPx, glutathione peroxidase; HSP, heat shock protein;HDL-C, high-density lipoprotein- cholesterol; HSP, heat shock protein;IFN, interferon;IGF-1, insulin-like growth factor 1; IL, interleukin; LP, lipid peroxidation; LDL-C, low-density lipoprotein- cholesterol; MetS, metabolic syndrome; Nrf2-ARE/HO-1, Nuclear Factor E2 related to Factor 2-antioxidant/heme oxygenase 1 response element;ROS/RNS, reactive oxygen species/reactive nitrogen species; SOD,superoxide dismutase; TNF, tumor necrose factor; Tregs, regulatory T cells; TrxR, thioredoxin reductase.

**Table references**

1. Tanacan, E. and N. Atakan, *Higher incidence of metabolic syndrome components in vitiligo patients: a prospective cross-sectional study☆,☆☆.* Anais brasileiros de dermatologia, 2020. **95**: p. 165-172.

2. Verma, D., et al., *Vitiligo: The Association With Metabolic Syndrome and the Role of Simvastatin as an Immunomodulator.* Cureus, 2021. **13**(3): p. e14029-e14029.

3. Ataş, H. and M. Gönül, *Increased Risk of Metabolic Syndrome in Patients with Vitiligo.* Balkan medical journal, 2017. **34**(3): p. 219-225.

4. Sharma, Y.K., et al., *Metabolic syndrome in vitiligo patients among a semi-urban Maharashtrian population: A case control study.* Diabetes & Metabolic Syndrome: Clinical Research & Reviews, 2017. **11**: p. S77-S80.

5. Namazi, N., et al., *Increased risk of subclinical atherosclerosis and metabolic syndrome in patients with vitiligo: a real association or a coincidence?* Dermatologic Therapy, 2021. **34**(2): p. e14803.

6. D’Arino, A., et al., *Metabolic Comorbidities in Vitiligo: A Brief Review and Report of New Data from a Single-Center Experience.* International Journal of Molecular Sciences, 2021. **22**(16): p. 8820.

7. Karadag, A.S., E. Tutal, and D.T. Ertugrul, *Insulin resistance is increased in patients with vitiligo.* Acta dermato-venereologica, 2011. **91**(5): p. 541-544.

8. Demirbaş, A., et al., *Can monocyte to HDL cholesterol ratio and monocyte to lymphocyte ratio be markers for inflammation and oxidative stress in patients with vitiligo? A preliminary study.* Archives of Dermatological Research, 2021. **313**(6): p. 491-498.

9. Azzazi, Y., et al., *Support for increased cardiovascular risk in non-segmental vitiligo among Egyptians: A hospital-based, case–control study.* Pigment Cell & Melanoma Research, 2021. **34**(3): p. 598-604.

10. Robinson, M.M., et al., *Enhanced Protein Translation Underlies Improved Metabolic and Physical Adaptations to Different Exercise Training Modes in Young and Old Humans.* Cell Metab, 2017. **25**(3): p. 581-592.

11. Lanza, I.R., et al., *Endurance exercise as a countermeasure for aging.* Diabetes, 2008. **57**(11): p. 2933-42.

12. Bird, S.R. and J.A. Hawley, *Update on the effects of physical activity on insulin sensitivity in humans.* BMJ Open Sport Exerc Med, 2016. **2**(1): p. e000143.

13. Jelleyman, C., et al., *The effects of high-intensity interval training on glucose regulation and insulin resistance: a meta-analysis.* Obesity Reviews, 2015. **16**(11): p. 942-961.

14. Fedewa, M.V., et al., *Exercise and Insulin Resistance in Youth: A Meta-Analysis.* Pediatrics, 2014. **133**(1): p. e163-e174.

15. Ostman, C., et al., *Clinical outcomes to exercise training in type 1 diabetes: A systematic review and meta-analysis.* Diabetes Research and Clinical Practice, 2018. **139**: p. 380-391.

16. Wewege, M.A., et al., *Aerobic, resistance or combined training: A systematic review and meta-analysis of exercise to reduce cardiovascular risk in adults with metabolic syndrome.* Atherosclerosis, 2018. **274**: p. 162-171.

17. da Silva, M.A.R., et al., *The Effects of Concurrent Training Combining Both Resistance Exercise and High-Intensity Interval Training or Moderate-Intensity Continuous Training on Metabolic Syndrome.* Frontiers in Physiology, 2020. **11**(572).

18. Su, L., et al., *Effects of HIIT and MICT on cardiovascular risk factors in adults with overweight and/or obesity: A meta-analysis.* PLOS ONE, 2019. **14**(1): p. e0210644.

19. Schallreuter, K.U., *Reactive Oxygen Species and Reactive Nitrogen Species in Vitiligo.* Systems Biology of Free Radicals and Antioxidants, 2014: p. 3697-3736.

20. Shajil, E. and R. Begum, *Antioxidant status of segmental and non‐segmental vitiligo.* Pigment cell research, 2006. **19**(2): p. 179-180.

21. Schallreuter, K.U., J.M. Wood, and J. Berger, *Low catalase levels in the epidermis of patients with vitiligo.* Journal of investigative dermatology, 1991. **97**(6): p. 1081-1085.

22. Maresca, V., et al., *Increased sensitivity to peroxidative agents as a possible pathogenic factor of melanocyte damage in vitiligo.* Journal of investigative dermatology, 1997. **109**(3).

23. Laddha, N.C., et al., *Involvement of superoxide dismutase isoenzymes and their genetic variants in progression of and higher susceptibility to vitiligo.* Free Radical Biology and Medicine, 2013. **65**: p. 1110-1125.

24. Barygina, V., et al., *Treatment with low-dose cytokines reduces oxidative-mediated injury in perilesional keratinocytes from vitiligo skin.* Journal of Dermatological Science, 2015. **79**(2): p. 163-170.

25. Laddha, N.C., et al., *Role of oxidative stress and autoimmunity in onset and progression of vitiligo.* Experimental Dermatology, 2014. **23**(5): p. 352-353.

26. Carlos Henríquez-Olguín, S.B., Claudio Cabello-Verrugio, Enrique Jaimovich, Elena Hidalgo, and Thomas E. Jensen, *The Emerging Roles of Nicotinamide Adenine Dinucleotide Phosphate Oxidase 2 in Skeletal Muscle Redox Signaling and Metabolism.* Antioxidants & Redox Signaling, 2019. **31**(18): p. 1371-1410.

27. Muthusamy, V.R., et al., *Acute exercise stress activates Nrf2/ARE signaling and promotes antioxidant mechanisms in the myocardium.* Free Radical Biology and Medicine, 2012. **52**(2): p. 366-376.

28. Aguiar, S.S., et al., *Master athletes have longer telomeres than age-matched non-athletes. A systematic review, meta-analysis and discussion of possible mechanisms.* Experimental Gerontology, 2021. **146**: p. 111212.

29. Margaritelis, N.V., et al., *Adaptations to endurance training depend on exercise-induced oxidative stress: exploiting redox interindividual variability.* Acta Physiologica, 2018. **222**(2): p. e12898.

30. Venditti, P., P. Masullo, and S. Di Meo, *Effect of Training on H2O2 Release by Mitochondria from Rat Skeletal Muscle.* Archives of biochemistry and biophysics, 1999. **372**(2): p. 315-320.

31. Qi, J., et al., *Swimming Exercise Protects against Insulin Resistance via Regulating Oxidative Stress through Nox4 and AKT Signaling in High-Fat Diet-Fed Mice.* Journal of Diabetes Research, 2020. **2020**: p. 2521590.

32. Vargas-Mendoza, N., et al., *Antioxidant and Adaptative Response Mediated by Nrf2 during Physical Exercise.* Antioxidants, 2019. **8**(6): p. 196.

33. Ding, G.-Z., et al., *A comparative study of mitochondrial ultrastructure in melanocytes from perilesional vitiligo skin and perilesional halo nevi skin.* Archives of dermatological research, 2015. **307**(3): p. 281-289.

34. Dell’Anna, M.L., et al., *Energetic mitochondrial failing in vitiligo and possible rescue by cardiolipin.* Scientific reports, 2017. **7**(1): p. 13663.

35. Dell'Anna, M.L., et al., *Membrane Lipid Alterations as a Possible Basis for Melanocyte Degeneration in Vitiligo.* Journal of Investigative Dermatology, 2007. **127**(5): p. 1226-1233.

36. Dell'Anna, M.L., et al., *Membrane lipid defects are responsible for the generation of reactive oxygen species in peripheral blood mononuclear cells from vitiligo patients.* J Cell Physiol, 2010. **223**(1): p. 187-93.

37. Dell'Anna, M.L., et al., *Alterations of mitochondria in peripheral blood mononuclear cells of vitiligo patients.* Pigment Cell & Melanoma Research, 2003. **16**(5): p. 553-559.

38. Bishop, D.J., J. Botella, and C. Granata, *CrossTalk opposing view: Exercise training volume is more important than training intensity to promote increases in mitochondrial content.* The Journal of physiology, 2019. **597**(16): p. 4115-4118.

39. Bishop, D.J., C. Granata, and N. Eynon, *Can we optimise the exercise training prescription to maximise improvements in mitochondria function and content?* Biochimica et Biophysica Acta (BBA)-General Subjects, 2014. **1840**(4): p. 1266-1275.

40. Das, S., et al., *ATP citrate lyase improves mitochondrial function in skeletal muscle.* Cell metabolism, 2015. **21**(6): p. 868-876.

41. Tarpey, M.D., et al., *Skeletal muscle autophagy and mitophagy in endurance-trained runners before and after a high-fat meal.* Molecular Metabolism, 2017. **6**(12): p. 1597-1609.

42. Hood, D.A., et al., *Exercise and the regulation of mitochondrial turnover.* Progress in molecular biology and translational science, 2015. **135**: p. 99-127.

43. Gram, M., et al., *Skeletal muscle mitochondrial H2O2 emission increases with immobilization and decreases after aerobic training in young and older men.* The Journal of Physiology, 2015. **593**(17): p. 4011-4027.

44. Lotti, T., J. Hercogova, and G. Fabrizi, *Advances in the treatment options for vitiligo: activated low-dose cytokines-based therapy.* Expert opinion on pharmacotherapy, 2015. **16**(16): p. 2485-2496.

45. Riding, R.L. and J.E. Harris, *The Role of Memory CD8<sup>+</sup> T Cells in Vitiligo.* The Journal of Immunology, 2019. **203**(1): p. 11-19.

46. Mosenson, J.A., *Defining a Role for Inducible Heat Shock Protein 70 (HSP70i) In Mediating Autoimmune Vitiligo.* 2013.

47. Turner, J.E., et al., *Intensive Exercise Does Not Preferentially Mobilize Skin-Homing T Cells and NK Cells.* Medicine and science in sports and exercise, 2016.

48. Moldoveanu, A.I., R.J. Shephard, and P.N. Shek, *The cytokine response to physical activity and training.* Sports medicine, 2001. **31**(2): p. 115-144.

49. Abd El-Kader, S., A. Gari, and A. Salah El-Den, *Impact of moderate versus mild aerobic exercise training on inflammatory cytokines in obese type 2 diabetic patients: a randomized clinical trial.* African health sciences, 2013. **13**(4): p. 857-863.

50. Hayashino, Y., et al., *Effects of exercise on C-reactive protein, inflammatory cytokine and adipokine in patients with type 2 diabetes: a meta-analysis of randomized controlled trials.* Metabolism, 2014. **63**(3): p. 431-440.

51. Minuzzi, L.G., et al., *Lifelong exercise practice and immunosenescence: Master athletes cytokine response to acute exercise.* Cytokine, 2019. **115**: p. 1-7.

52. Alizaei Yousefabadi, H., et al., *Anti-Inflammatory Effects of Exercise on Metabolic Syndrome Patients: A Systematic Review and Meta-Analysis.* Biological Research For Nursing, 2020. **23**(2): p. 280-292.

53. Chen, Z., et al., *Upregulation of IL-4 signaling contributes to aerobic exercise-induced insulin sensitivity.* Biochemical and Biophysical Research Communications, 2020. **525**(3): p. 662-667.

54. Souza, P.S., et al., *Physical Exercise Attenuates Experimental Autoimmune Encephalomyelitis by Inhibiting Peripheral Immune Response and Blood-Brain Barrier Disruption.* Molecular Neurobiology, 2017. **54**(6): p. 4723-4737.

55. Rhind, S., et al., *Differential expression of interleukin-2 receptor alpha and beta chains in relation to natural killer cell subsets and aerobic fitness.* International journal of sports medicine, 1994. **15**(06): p. 311-318.

56. Mooren, F.C. and K. Krüger, *Exercise, autophagy, and apoptosis.* Progress in molecular biology and translational science, 2015. **135**: p. 407-422.

57. Shiu, M., *Modulation of T Cell Distribution and Function by High-Intensity Interval Training*. 2016, University of Toronto.

58. Minuzzi, L.G., et al., *Effects of lifelong training on senescence and mobilization of T lymphocytes in response to acute exercise.* Exercise immunology review, 2018. **24**.

59. Donovan, T., et al., *Influence of Exercise on Exhausted and Senescent T Cells: A Systematic Review.* Frontiers in Physiology, 2021: p. 1331.

60. Minuzzi, L.G., et al., *Lifelong training improves anti-inflammatory environment and maintains the number of regulatory T cells in masters athletes.* 2017.

61. Weinhold, M., et al., *Physical exercise modulates the homeostasis of human regulatory T cells.* Journal of Allergy and Clinical Immunology, 2016. **137**(5): p. 1607-1610. e8.

62. Proschinger, S., et al., *The effect of exercise on regulatory T cells: A systematic review of human and animal studies with future perspectives and methodological recommendations.* Exercise Immunology Review, 2021. **27**: p. 142-166.

63. Périard, J.D., et al., *Cardiovascular adaptations supporting human exercise-heat acclimation.* Autonomic Neuroscience, 2016. **196**: p. 52-62.
